# Electron-Poor Phosphines Enable the Selective Semihydrogenation Reaction of Alkynes with Pd on Carbon Catalysts

**DOI:** 10.1021/acs.jpclett.2c03428

**Published:** 2023-01-23

**Authors:** Jordi Ballesteros-Soberanas, Antonio Leyva-Pérez

**Affiliations:** Instituto de Tecnología Química (UPV-CSIC), Universidad Politècnica de València-Consejo Superior de Investigaciones Científicas, Avda. de los Naranjos s/n, 46022 Valencia, Spain

## Abstract

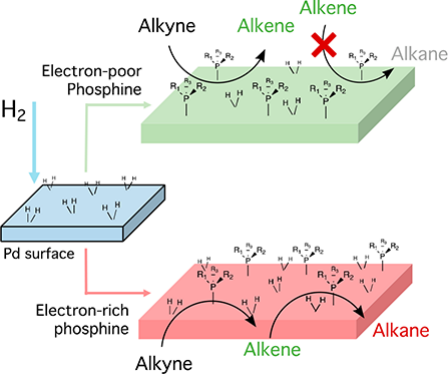

An alternative to the Lindlar catalyst for the semihydrogenation reaction of alkynes to alkenes is of high interest. Here we show that palladium on carbon (Pd/C), i.e., a widely available supported Pd catalyst, is converted from an unselective to a chemoselective catalyst during the semihydrogenation reaction of alkynes, after the addition of catalytic amounts of commercially available electron-poor phosphines. The catalytic activity is ≤7 times greater, and the selectivity is comparable to that of the industrial benchmark Lindlar catalyst.

The Pd-catalyzed selective semihydrogenation of alkynes to alkenes is a key industrial reaction for preparing *cis*-alkenes in the easiest way. These alkenes are utilized in the synthesis of nutraceuticals, pheromones, vitamins, etc.^1^ Simple catalysts consisting of supported bare Pd nanoparticles are not selective, including the widely commercially available palladium on carbon solid (Pd/C). Consequently, catalytic Pd nanoparticles (NPs) must be modified to be selective, such as, for instance, in the classical Lindlar catalyst, composed of PdPb NPs supported on CaCO_3_, often selectively poisoned with quinoline.^2^

Alloying or decorating the active Pd phase with other metals is a common practice in alkyne semihydrogenation reaction catalysts when trying to enhance the selectivity toward the alkene.^3−6^ The metal surface has been selectively poisoned to energetically favor the desired reaction or/and suppress the undesired reactions, i.e., the use of quinolines on the Lindlar catalyst.^7^ Other poisoning agents such as sulfides have been successfully used to modify the hydrogenation selectivity,^8^ by depositing sulfur on the Pd surface,^9,10^ using sulfides as a support,^11,12^ or combining both the palladium sulfide surface with thiol modifiers.^13^ Nitrogen doping of the catalyst near the active Pd sites,^14,15^ Au sites,^16,17^ and Co sites^18^ has also been proposed, as well as a more recently reported dynamic adsorption control from alkylamino chains over the Pd NPs,^19^ which was similarly performed previously with sulfur-containing groups.^20,21^ In a similar fashion, phosphorus has been identified as a beneficial additive for alkyne semihydrogenation reactions, as a support itself,^22,23^ in a phosphine-functionalized polymer surrounding supported Pd NPs,^24^ in phosphines on supported Pd NPs,^25,26^ or as stabilizers of colloidal Pd NPs.^27^ This latter approach has been employed on other metals such as Ru^28^ or Rh.^29^ However, the use of commercially available Pd/C modified with simple phosphine modifiers as a catalyst for the semihydrogenation reaction of alkynes has, to the best of our knowledge, not been studied yet, despite the abundance of accessible commercial phosphines and the widespread use of these ligands for organometallic complexes.

Structure–activity relationship (SAR) studies have been performed for phosphine metal catalysts in a variety of reactions, parametrized by the steric and electronic properties of the ligands.^30^ In this work, in addition to studying the catalytic effect produced by the addition of phosphines on Pd/C to the alkyne semihydrogenation reaction mixture, we delve into the interaction between free phosphines and the supported Pd NPs to establish correlations between the structure and properties of free phosphine ligands and their effects on reaction rates and selectivity for the reaction. In this way, we will show the optimal conditions for a selective hydrogenation with Pd/C, a well-known unselective catalyst for semihydrogenation reactions.

Phosphines with diverse properties were selected (Table 1), and their effect on the hydrogenation of 3-methyl-1-pentyn-3-ol (**1**) to the corresponding alkene (**2**) with the Pd/C catalyst (0.01 mol %) was studied (Figure 1). The selected phosphines can be classified into five categories: symmetric P–N ligand phosphines (PX_3_), symmetric P–C ligand phosphines (PR_3_), asymmetric P–C ligand phosphines [P(R^1^)_2_R^2^], diphosphines (P_2_R_4_), and Buchwald type phosphines. The different substituents cover a wide range of steric and electronic properties, with cone angles ranging from 127° to 205°,^31,32^ as well as a wide array of electron-accepting and -donating capabilities. The latter is parametrized as the vibrational frequency of the carbonyl stretch of the corresponding Ni(CO)_3_L complex^31,32^ (2056.1–2073.0 cm^–1^), which correlates to the phosphine lone pair charge density.^33^ The individual substituent contributions and the calculation of the stretch frequencies can be found Table S1. Different commercial samples of Pd/C catalysts were used as received, and high-resolution scanning transmission electron microscopy (HR-STEM) imaging reveals a broad particle size distribution, with most of the palladium species being smaller than 15 nm (Figure S1).

**Table 1:** Electronic Properties, Expressed as the Carbonyl Vibrational Frequency of the Corresponding Ni(CO)_3_(PR_3_) Complex, and Cone Angles Formed by the Phosphine Substituents in Complexes **P1–P11** Used in This Work

type	number	phosphine	Ni(CO)_3_L ν(CO) (cm^–1^)	Tolman cone angle^f^ (deg)
PX_3_	**P1**	P(NEt_2_)_3_	2061.8^a^ ± 0.3	157.0
PR_3_	**P2**	P(^*t*^Bu)_3_	2056.1 ± 0.3	182.0
	**P3**	PCy_3_	2056.4 ± 0.3	170.0
	**P4**	PPh_3_	2069.0 ± 0.3	145.0
P(R^1^)_2_R^2^	**P5**	P(^*t*^Bu)_2_Me	2058.7 ± 0.3	161.0
	**P6**	PCy_2_H	2064.6 ± 0.3	142.3
	**P7**	PPh_2_H	2073.0 ± 0.3	128.0
P_2_R_4_	**P8**	bis(PPh_2_)Ph	2066.9^b^ ± 0.3	127.0^g^
	**P9**	bis(PPh_2_)Pr	2065.6^c^ ± 0.3	127.0
Buchwald	**P10**	SPhos	2059.7^d^ ± 0.3	204.4^h^
	**P11**	JohnPhos	2060.4^e^ ± 0.3	184.1^h^

a Estimated as NMe_2_.

b Estimated as Ph/2.

c Estimated as Et/2.

d Estimated as (*o*-C_6_H_4_OMe).

e Estimated as Ph.

f From ref (31).

g Assumed to have the same angle as **P9**.

h From ref (32).

**Figure 1:**
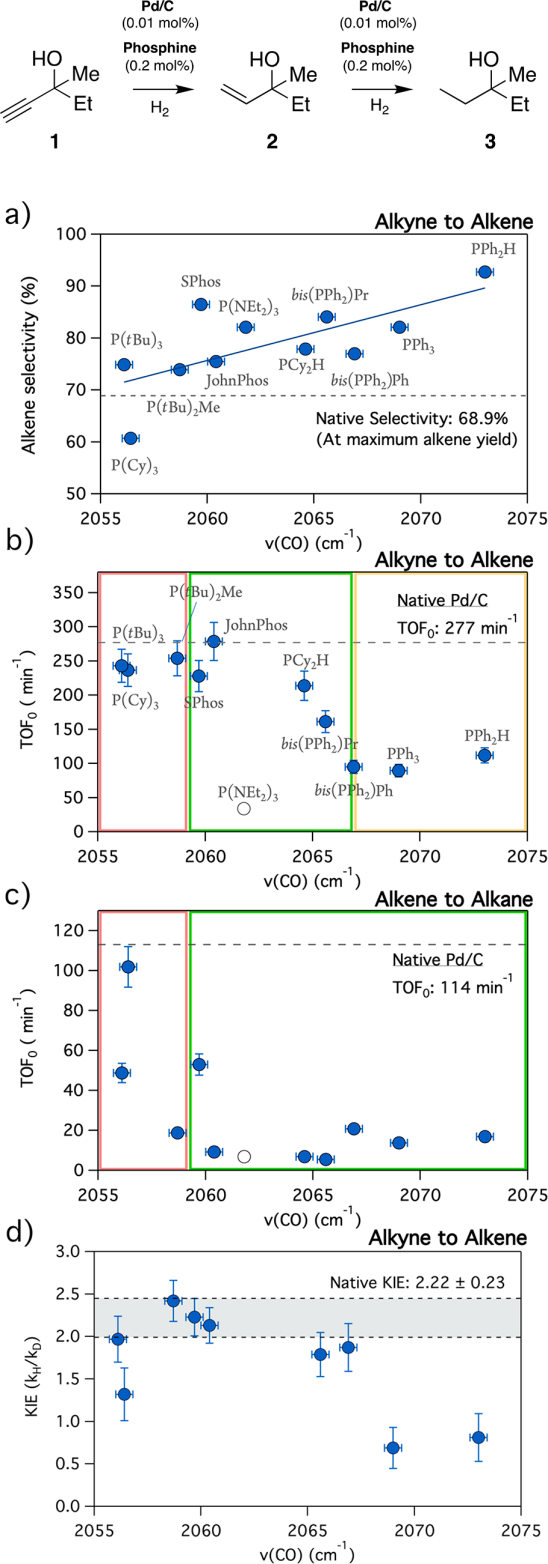
Correlations of (a) selectivity toward the alkene (**2**) in the hydrogenation of **1**, (b) initial turnover frequencies in the alkyne to alkene (**1** → **2**) and (c) alkene to alkane (**2** → **3**) hydrogenation reactions, and (d) the kinetic isotope effect (KIE) in the hydrogenation reaction of **1**, as a function of the phosphine electronic properties, expressed in terms of the ν(CO) (cm^–1^) of the corresponding Ni(CO)_3_(PR_3_) complex. The reactions were performed with Pd/C (0.01 mol % Pd) at 3 bar, with a 1:20 Pd:phosphine ratio, in duplicate; thus, the results are an average. In the red areas, **2** can be hydrogenated under these conditions. In the yellow areas, **2** cannot be hydrogenated and we find low hydrogenation rates of **1**. In the green areas, **2** cannot be hydrogenated and we find high hydrogenation rates of **1**. Horizontal error bars represent the ±0.3 cm^–1^ uncertainty reported by Tolman et al.

Figure 1 shows the results for the hydrogenation reaction of alkyne **1** catalyzed by Pd/C (0.01 mol %) under 3 bar of H_2_ and with different phosphines as ligands, added in 20-fold excess with respect to the catalyst to ensure a full coverage. The obtained selectivities and intrinsic initial rates have been plotted as a function of the phosphine inductive strength, and the numerical values of the catalytic turnovers are listed in Table S2. To further understand the effect of the phosphine in each step of the hydrogenation process, the hydrogenation of alkene **2** to its corresponding alkane was also carried out separately (Figure 1c), under the same conditions. In addition, the hydrogenation of alkyne **1** was also performed with D_2_ to assess the effect of each individual phosphine on the kinetic isotope effect (KIE) (Figure 1d).

Figure 1a shows that upon addition of the phosphines to the Pd/C catalyst, the selectivity of the hydrogenation reaction of **1** toward alkene **2** increases, compared to the selectivity of the bare solid catalyst. A clear, linear dependence between the selectivity and the phosphine electron-donating ability can be drawn, where less donating phosphines [higher ν(CO) values] maximize the selectivity enhancement, and the more donating phosphines [lower ν(CO) values] struggle to palliate the overhydrogenation reaction. The selectivities correspond to the values at the maximum alkene yield (>99% in many cases), and these selectivities are generally maintained at longer reaction times by the more electrophilic phosphines, as observed in the corresponding full kinetic profile (Figures S6–S8). Simultaneously, panels b and c of Figure 1 show that the alkyne and alkene hydrogenation initial rates (expressed as initial turnover frequencies, TOF_0_) decrease in the presence of some phosphine ligands, when compared to those of the Pd/C native catalyst. In particular, the only amine-containing phosphine (**P1**) displays an almost complete inhibition for all reactions. The strong dependence between the poisoning capability of the phosphine additives and their electron-donating character is expressed as a sigmoidal curve, with a reaction onset at 2067.5 cm^–1^ for the hydrogenation reaction of alkyne **1**, while the hydrogenation reaction of alkene **2** presents the onset at 2060.4 cm^–1^.

Moreover, one can see that while the reaction rates of **2** to **3** become zero after the onset, at lower phosphine inductive strengths [higher ν(CO) frequencies], the rates of **1** to **2** never decrease to zero after the onset, regardless of the conditions. In other words, when the phosphine achieves its maximum impact on the catalyst [high ν(CO) frequencies], it hinders but does not preclude the hydrogenation of the alkyne, while the alkene hydrogenation can be fully suppressed.

In an effort to elucidate the source of the promoting effects of the phosphines on the hydrogenation reaction, several experiments were performed with PPh_3_ (**P4**) and S-Phos (**P10**) to investigate the interactions between (i) the Pd surface and the phosphines, (ii) the phosphines and the surface hydrides, and (iii) the phosphines and the reaction substrate. First, a leaching test revealed that the catalytically active species remained on the solid after the addition of S-Phos and that migration to the liquid phase does not occur regardless of the phosphine equivalents employed (Figure S2). Inductively coupled plasma adsorption emission spectrophotometry (ICP-AES) analysis of the reaction media confirmed the absence of palladium in the liquid phase. ^31^P solid state nuclear magnetic resonance showed the presence of phosphorus on the catalyst, after the Pd/C catalyst had been mixed with 1 equiv of PPh_3_. The chemical shift of the phosphorus, from −8.9 ppm (free PPh_3_) to 18.5 ppm, indicates the bonding of the phosphine to Pd (Figure S3), thus confirming the interaction between the phosphine and the metal surface, which occurs on the heterogeneous catalyst according to the leaching test. Second, the phosphorus elemental analysis (ICP-AES), performed on solutions containing Pd/C catalysts with PPh_3_ (1:1 P:Pd ratio), confirmed that the phosphines are not displaced during reaction, regardless of the H_2_ pressure used [0–7 bar H_2_ (Figure S4)]. Raman spectroscopy was employed to further assess the effect of PPh_3_ on the Pd–H bond, and any shift was not observed in the encountered Pd–H bands, confirming that the phosphine does not affect the formation of metal–hydride bonds on the metal nanoparticle (Figure S5). The persistence of phosphines coordinated to the Pd surface during reaction, probably through their lone pairs and without any oxidation, precludes their participation in a hypothetical heterolytic H_2_ bond cleavage.^34^ Third, after confirming the Pd–P interaction and ruling out the phosphine–hydride interaction, we focused on alkyne adsorption. Figure 1d shows that the rate-determining step of the hydrogenation of **1** on the surface of the bare Pd/C catalyst is the H–H cleavage (*k*_H_/*k*_D_ = 2.2), but in the presence of the phosphines, the KIE decreases as a function of the electron donating capability of the phosphine, in a fashion similar to the decrease in the initial turnover values presented in Figure 1b. Hence, given the fact that the most energetically demanding step in the reaction is hydrogen splitting, in the absence of phosphines, and that electron-poor phosphines such as PPh_3_ do not ease the cleavage (Figure S5) but decrease the KIE values to ∼1, we must conclude that the step that precedes H_2_ dissociation, i.e., the adsorption of the alkyne, is the limiting step of the Pd/C phosphine-catalyzed reaction, hampered by electron-poor phosphines.

The adsorption of alkynes and alkenes on Pd surfaces has been extensively studied, and it is well-known that the former is more exothermic.^2^ Thus, if the alkyne adsorption step limits the hydrogenation reaction, a difference is to be expected in the effects of the phosphines on the alkyne and alkene hydrogenation reactions, which is observed as a shift of ∼7 cm^–1^ in the aforementioned reaction onsets of **1** and **2** (Figure 1b,c), in terms of ν(CO). Closer analysis of the individual kinetic profiles for the hydrogenation reaction of both **1** and **2** with electron-poor phosphines (Figures S6–S8) revealed that the formation of alkane **3** can be detected only when alkyne **1** is used as a reactant. When phosphines with ν(CO) frequencies higher than the onset ν(CO) frequency are used, alkane **3** is not formed from **2** in solution, therefore supporting the idea that the adsorption of alkene **2** from the liquid phase is prevented.

These findings indicate that the use of the right phosphine, as shown by the colored areas in Figure 1, enables the selective semihydrogenation of alkyne **1** to alkene **2** with the unselective Pd/C catalyst. Figure 2 shows that S-Phos provides a relevant improvement in the suppression of alkane formation at longer reaction times, while the selectivity at the maximum alkene yield increases to 82%, without any activity loss. Despite other systems having been studied with similar increases in selectivity,^24,35,36^ the ability of the Pd/C/phosphine catalyst to achieve a high alkene selectivity without losing catalytic activity is rather unique. To put the results in perspective, the Pd/C/S-Phos catalytic system significantly improves the results of the Lindlar catalyst, a staple of selective alkyne semihydrogenation reactions, by achieving the same results but using 7 times less catalytically supported palladium metal (Figure 2b). Moreover, the Lindlar catalyst benefits too from the addition of S-Phos, increasing the maximum selectivity of the reaction from ∼82% (unmodified) to 95.2% (S-Phos-modified). The performance of this promoter was compared to that of quinoline, and it was found that while the increase in maximum selectivity at full conversion is similar for both, the reaction rate is much higher in the presence of S-Phos, indicating a more moderate and efficient poisoning effect (Figure 2c).

**Figure 2:**
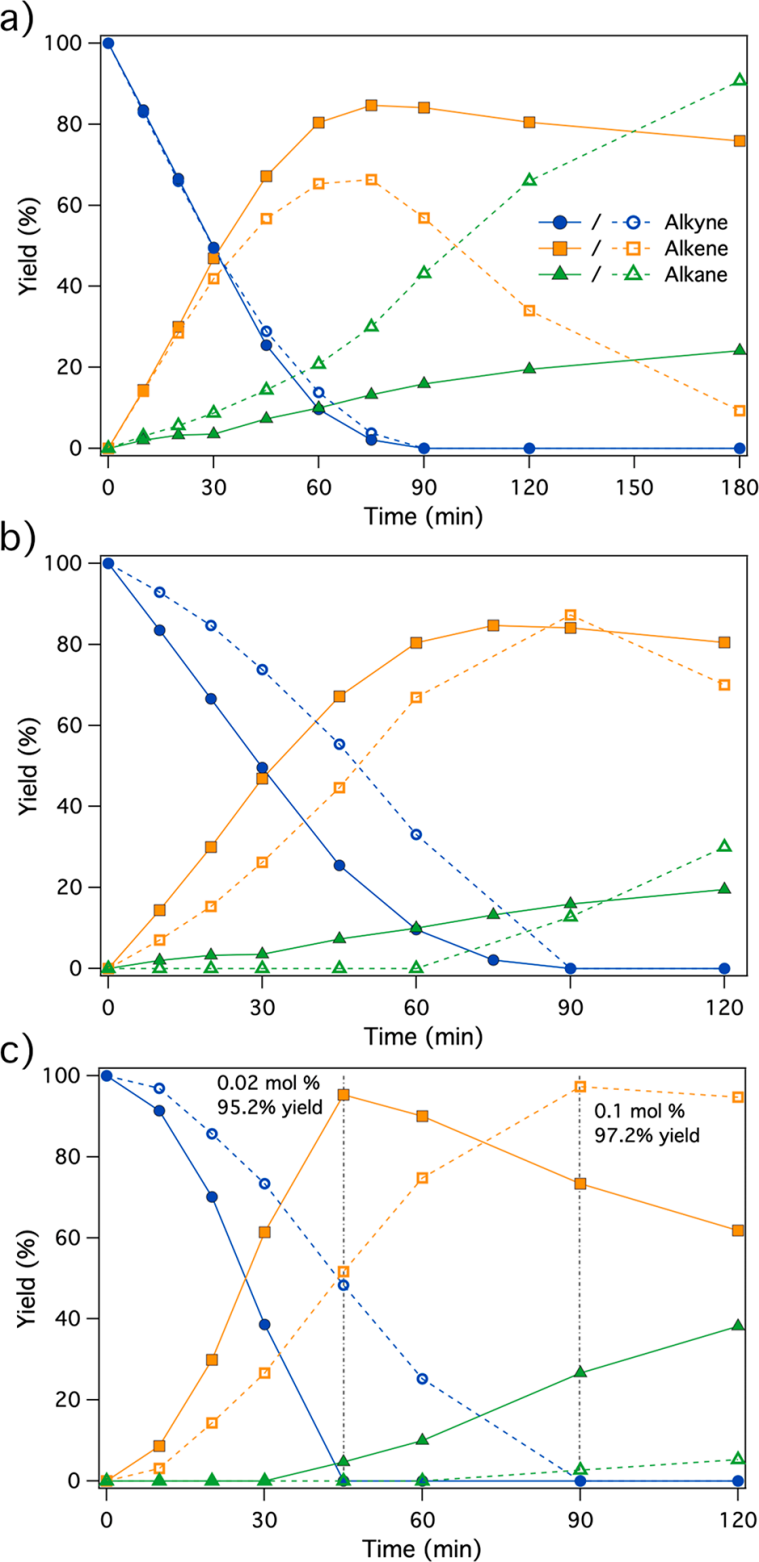
(a) Kinetic profiles for the S-Phos-modified (20:1 S-Phos:Pd ratio, filled symbols) and unmodified (empty symbols) during the hydrogenation of **1** under 3 bar of H_2_ with Pd/C (0.01 mol % Pd). (b) Kinetic profiles for the S-Phos-modified Pd/C catalyst (0.01 mol % Pd, 20:1 S-Phos:Pd ratio, filled symbols) and unmodified Lindlar catalyst (0.07 mol % Pd, empty symbols) during the hydrogenation of **1** under 3 bar of H_2_. (c) Kinetic profiles for the S-Phos-modified (20:1 S-Phos:Pd ratio, 0.02 mol % Pd, filled symbols) and quinoline-modified (20:1 quinoline:Pd ratio, 0.1 mol % Pd, empty symbols) Lindlar catalyst during the hydrogenation of **1** under 3 bar of H_2_.

To validate and extend the applicability of the phosphine-modified hydrogenation reactions with the Pd/C catalyst, 1-octyne (**4**) and phenylacetylene (**5**), typical model aliphatic and aromatic terminal alkynes, respectively, were studied following the same methodology. The hydrogenation rates of compound **1** in the presence of phosphines were also determined under 1, 3, and 5 bar of H_2_ and under 3, 5, and 7 bar of H_2_ for **4** and **5**, with phosphines **P3**–**P5**, **P7**, **P8**, and **P11**, respectively. These phosphines were selected to include phosphines of each substituent category in the study, while maintaining a wide range of ν(CO) vibrational frequencies (2056.4–2073.0 cm^–1^). Figure 3 shows the correlation between the substrate reaction rates and the phosphine electronic properties, and the selectivity results under 3 bar of H_2_. This study strongly supports the idea that the nonlinear dependence of the hydrogenation rates with the electron-donating character of the phosphine is ubiquitous, regardless of the hydrogenated alkyne (Figure 3a–c) and that the selectivity can be improved for different alkynes other than **1** (Figure 3d). For alkynes **1** and **4**, higher H_2_ pressures were found to require less donating, more restrictive phosphine ligands to sufficiently hinder the alkene adsorption, while this effect was not appreciated for alkyne **5**. However, it must be noted here that while alkene adsorption is still prevented, the selectivity decreases at high H_2_ pressures (>5 bar), which we tentatively attribute to the immediate hydrogenation of the alkene on the surface upon alkyne hydrogenation and before desorption, exacerbated by relatively high H_2_ pressures (Figures S8 and S9). In contrast with recently published results for colloidal Pd NPs,^27^ we did not find a volcano plot, but a linear correlation between the turnovers and the cone angles, with the higher rates being observed with larger cone angle phosphines (Figures S10–S12). We did not find any significant correlation between the cone angle and the semihydrogenation reaction selectivity.

**Figure 3:**
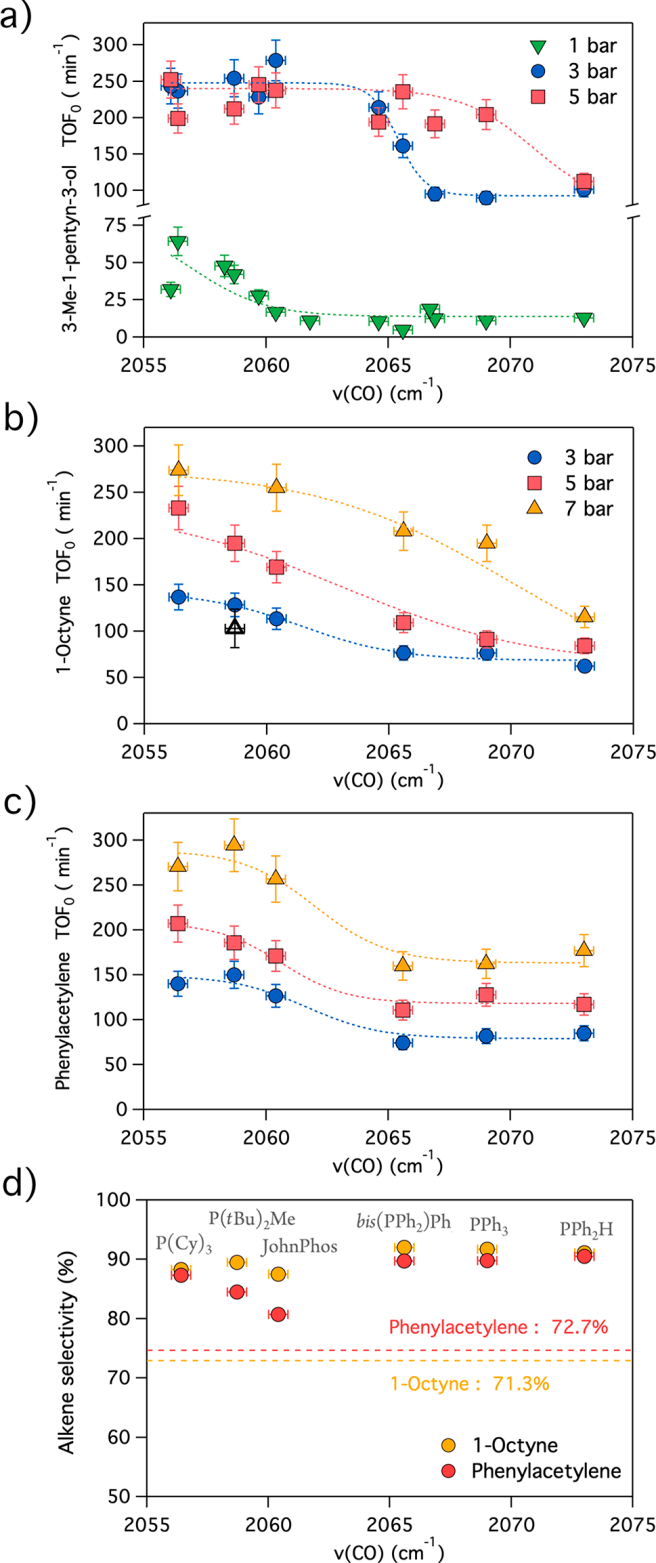
Correlations of the initial turnover frequencies in the alkyne to alkene hydrogenation reactions of (a) 3-methyl-1-pentyn-3-ol (**1**), (b) 1-octyne (**4**), and (c) phenylacetylene (**5**), as a function of the phosphine electronic properties, expressed in terms of the ν(CO) (cm^–1^) of the corresponding Ni(CO)_3_(PR_3_) complex. The sigmoidal dashed lines are a guide to the eye. (d) Selectivity at the maximum alkene yield of the hydrogenation reactions of **4** and **5** under 3 bar of H_2_ with phosphines **P3**–**P5**, **P7**, **P8**, and **P11**. The dashed horizontal lines indicate the corresponding selectivity of the unmodified Pd/C catalyst. The reactions were performed with Pd/C (0.01 mol %) at 3 bar of H_2_ and a 1:20 Pd:phosphine ratio.

In conclusion, we have found that the semihydrogenation reaction of alkynes to alkenes can be catalyzed under moderate H_2_ pressures (<5 bar) with Pd/C catalysts and phosphines as additives, both commercially available, and the results are better than those achieved by the Lindar catalyst under the same reaction conditions. Indeed, the phosphine-modified catalysts can be reused up to four times without decreases in the catalytic activity or selectivity promoting effect in the case of PPh_3_, and with <5% losses from use to use in the case of S-Phos (Figure S13). It is worth noting here that the addition of more phosphine between reuses is not required.

## Supporting Information Available

Experimental methods, TEM catalyst images, leaching test, ICP-AES data, Raman spectroscopy, individual kinetic profiles, phosphine cone angle effects on hydrogenation rates, and reuse tests (Figures S1–S13) and contributions of the phosphine ligand to electronic and steric parameters and TOFs for hydrogenation reactions of alkyne **1** (Tables S1 and S2, respectively) (PDF)
