# Supplementary material for: Electron-Poor Phosphines Enable the Selective Semihydrogenation Reaction of Alkynes with Pd on Carbon Catalysts
Source: J Phys Chem Lett. 2023 Jan 23;14(4):965–70. doi: 10.1021/acs.jpclett.2c03428 (PMC9900635; doi:10.1021/acs.jpclett.2c03428)
Supplement: Supplementary file 1 — jz2c03428_si_001.pdf [file jz2c03428_si_001.pdf]

SUPPORTING INFORMATION

Electron Poor Phosphines Enable the Selective Semi-hydrogenation Reaction of Alkynes with Pd on Carbon Catalysts

Jordi Ballesteros-Soberanas,[a] and Antonio Leyva-Pérez.*[a]

[a] Instituto de Tecnología Química (UPV-CSIC), Universidad Politècnica de València-Consejo Superior de Investigaciones Científicas, Avda. de los Naranjos s/n, 46022 Valencia, Spain.

Corresponding author e-mail: anleyva@itq.upv.es

<u>Table of contents</u>

**Experimental Methods**	p. S2
**Supporting Figures** (Figures S1 – S13)	p. S3-S20
**Supporting Tables** (Table S1-S2)	p. S21-S22
**References**	p. S23

Experimental Methods

Materials. Alkynes **1, 4** and **5** and phosphines were purchased from Merck-Millipore Sigma (95–99% purity), except for **P6** (10% in hexane) and **P10** (98% purity), purchased from Abcr. Any other chemicals were purchased and used as received. Pd/C (1 wt%) was purchased from Merck-Millipore Sigma, Alfa Aesar and Thermo Fisher Scientific, and used as received. Pd/C 1 wt% from Merck-Millipore Sigma was used for most reactions in this study.

Synthesis. Alkene **2** was independently synthesized by selectively hydrogenating **1** under 1 bar of H_2 with colloidal Pd nanoparticles supported on TiS (c-Pd/Tis, BASF) at room temperature. Two batch of 1 g each were produced in 15 mL ethanol, with full alkene conversion and 10% (for batch 1) and 20% (for batch 2) of alkane contents in the final product mixture.

Hydrogenation reactions. All the batch reactions in this work were performed in a 6 mL round bottom vial, with 0.5 mL of reaction volume and a stirring magnet. Solvent was ethanol, at 0.3 M concentration when the pressure was set at 1 bar of H_2, and at 0.6 M concentration under higher pressures of H_2. The reactions were conducted at room temperature and stirred at 450 rpm. The H_2 pressure was kept constant by refilling the reactor periodically, so enough H_2 was always present to fully hydrogenate the alkyne to the corresponding alkane. Yields were obtained by gas chromatography, and GC-coupled mass spectrometry and NMR were used to identify the products, besides comparison with pure product samples

Reuses: Reactions were performed and analyzed following the above procedure; conditions: 3 bar H_2, 0.6 M **1** and 30 ºC. After reaction, the solid catalyst was recovered by centrifugation (3000 rpm), washed with ethanol and centrifugated again, and weighed for the next use.

Electron microscopy characterization. Images of the Pd/C catalyst samples were obtained on a JEM-F2100 operated at 200 kV in Dark Field Scanning Transmission Electron Microscopy (DF-STEM mode).

NMR. Solid state ^{31}P MAS NMR of the Pd/C sample containing PPh_3 was recorded in a Bruker Ascend 400WB, with a 90 pulse of 4 μs and a recycle delay of 20 s, spinning the sample at 20 kHz. The spectrum was referenced to H_3PO_4 (0 ppm).

Elemental Analysis. The metal content of the samples was determined by inductively coupled plasma-adsorption emission spectrophotometry (ICP−AES). Solids were disaggregated in *aqua regia* and later diluted in miliQ water before analysis. The Pd/C digestion solutions were centrifuged and filtered prior to analysis.

Raman spectroscopy. The Raman spectra of the Pd/C and Pd/C + PPh_3 catalysts were obtained with an excitation wavelength of 514 nm in a Renishaw inVia spectrometer, under a stream of H_2.

Supporting Figures

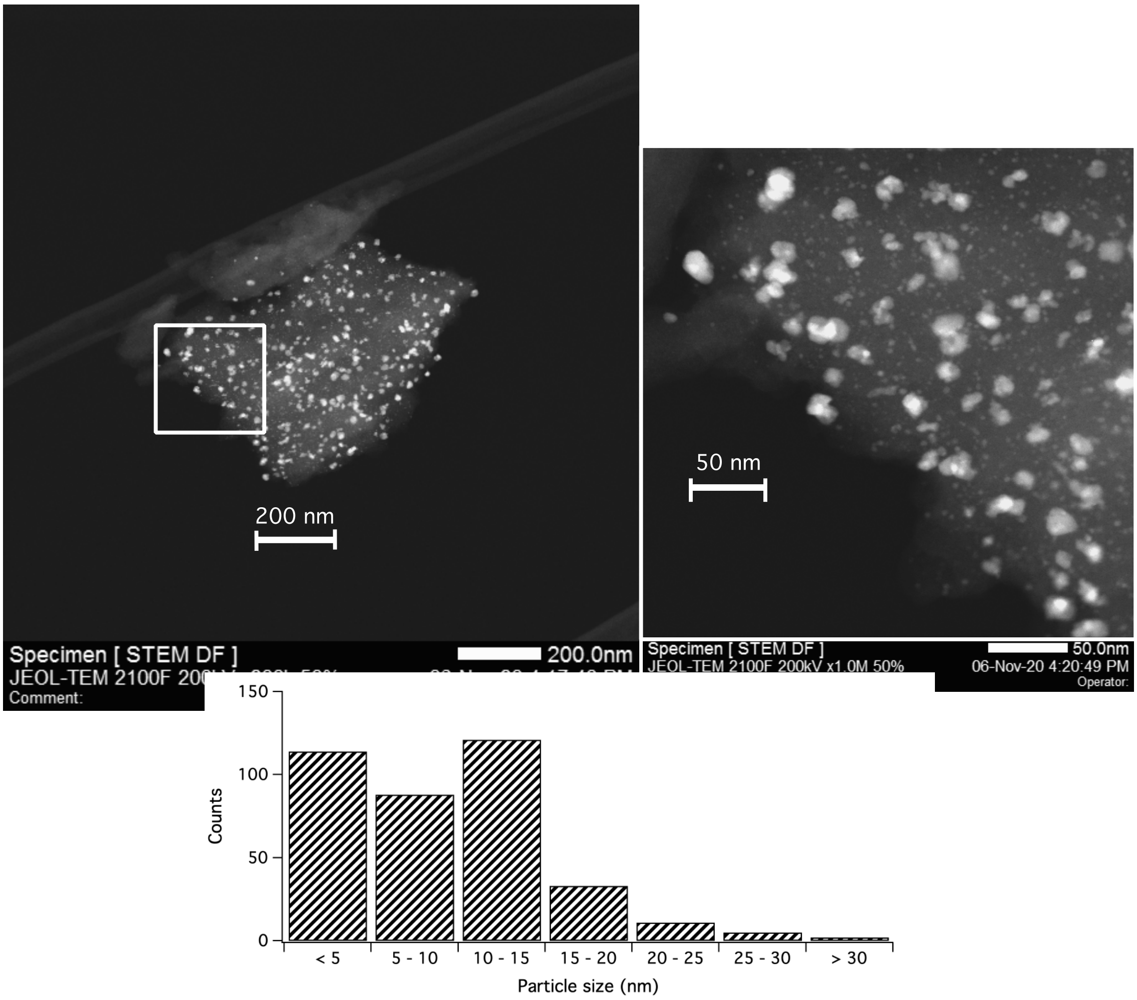

Figure S1. Dark field STEM images of a representative commercial Pd on carbon sample (1 wt%) from Merck-Millipore Sigma.

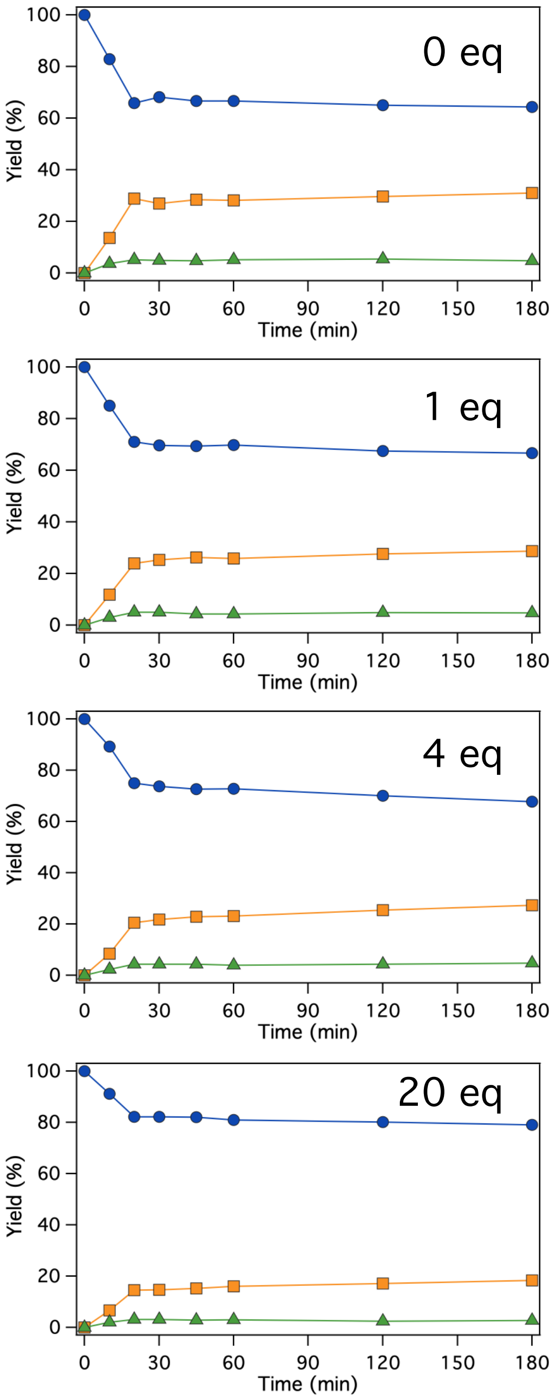

Figure S2. Leaching tests at 0, 1, 4 and 20 equivalents of SPhos to Pd/C (0.01 mol%) in the hydrogenation of **1** under 3 bar of H_2. Blue = alkyne (**1**), orange = alkene (**2**), green = alkane (3).

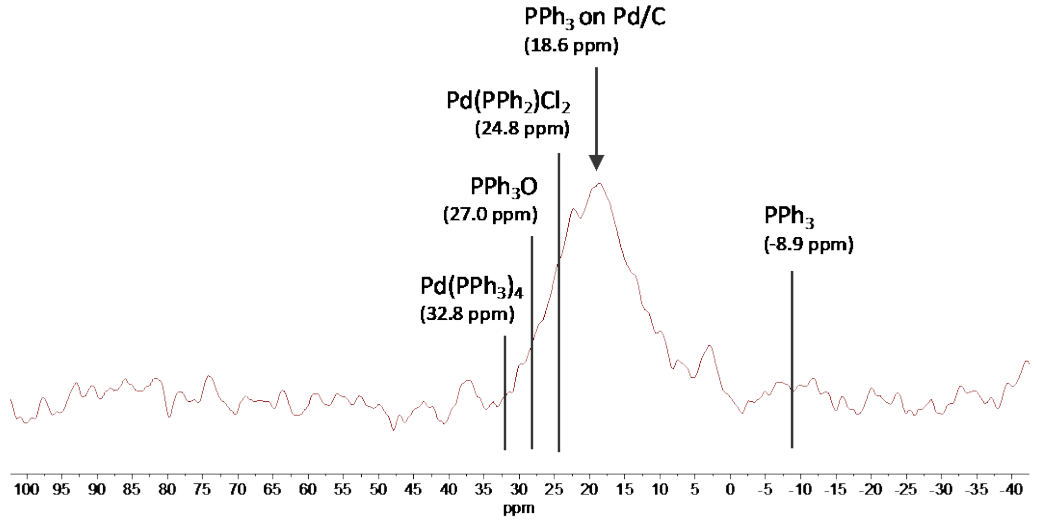

Figure S3. ^{31}P solid state MAS NMR of the Pd/C catalyst mixed with 1 equivalent of triphenylphosphine (PPh_3). The catalyst and the phosphine were stirred together for 1 h in ethanol, and the catalyst was afterwards centrifugated and separated, and further rinsed with cold ethanol. A new peak was observed in the spectrum at 18.6 ppm. The bibliographic values for the ^{31}P chemical shifts of PPh_3[S1] (solid-state NMR), $Pd(PPh_2)Cl_2$[S1] (solid-state NMR), $Pd(PPh_3)_4$[S2] (in $CDCl_3$) and PPh_3O[S3] (in $CDCl_3$) have been included for comparison.

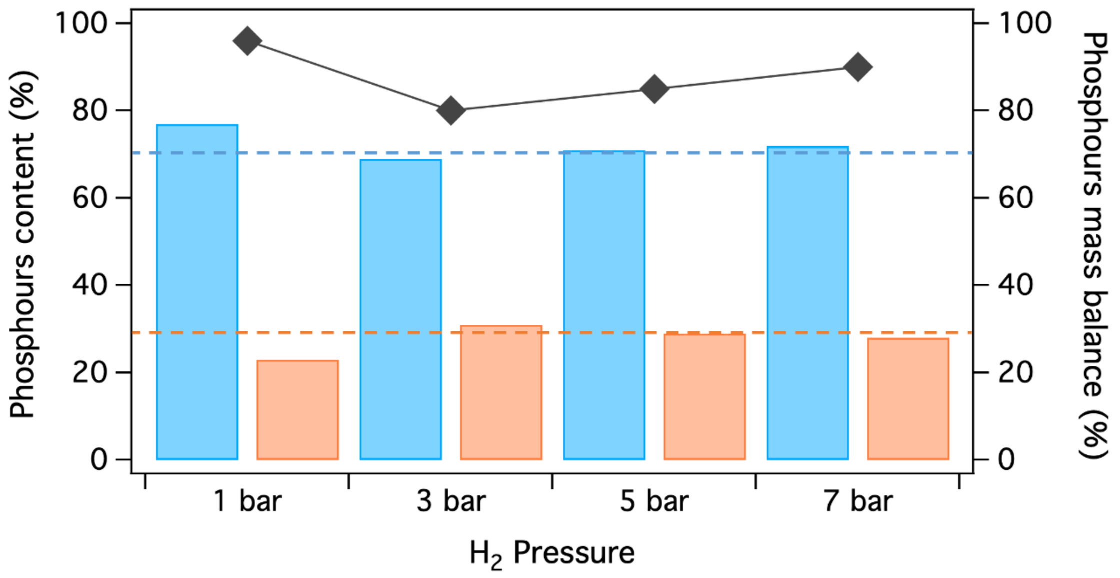

Figure S4. Phosphorus elemental analysis (ICP-AES) of the ethanolic reaction solution containing Pd/C + PPh_3 (1:1 P to Pd, blue), under different H_2 pressures. Dashed lines indicate the baseline values for the P content in the experiment, performed in ethanol, in absence of H_2. The solid (orange) and liquid (blue) phases of the reaction mixtures were separated by sedimentation in order to maintain the pressure inside the reactor. After 24 h, the solvent was removed through the sampling capillary tube of the reactor, and the remaining solid and collected liquid phases were dried under vacuum, and digested with *aqua regia* + H_2O_2. The digested solid phases were filtered to remove the non-disaggregated carbonaceous residues before elemental analysis.

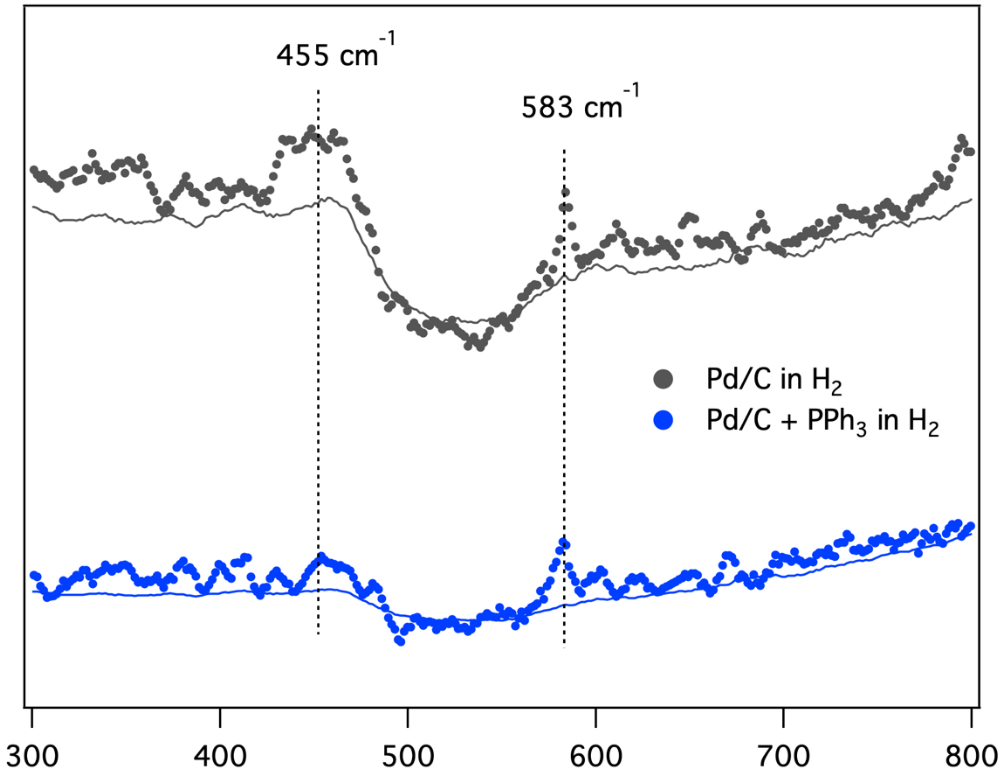

Figure S5. Raman spectroscopy of the Pd on carbon (Pd/C) catalyst and the Pd/C on catalyst mixed with 1 equivalent of triphenylphosphine (PPh_3) in presence of H_2. The catalyst and the phosphine were stirred together for 1 h in ethanol, and the catalyst was afterwards centrifugated and separated, and further rinsed with cold ethanol. The shape and separation between both maxima (128 cm^{-1}) is in good agreement with bibliographical data for Pd-H, positions of the maxima are shifted 17 cm^{-1} with respect to the reported values from Sherman et al.[S4] at ultra-low temperatures (472 and 610 cm^{-1}).

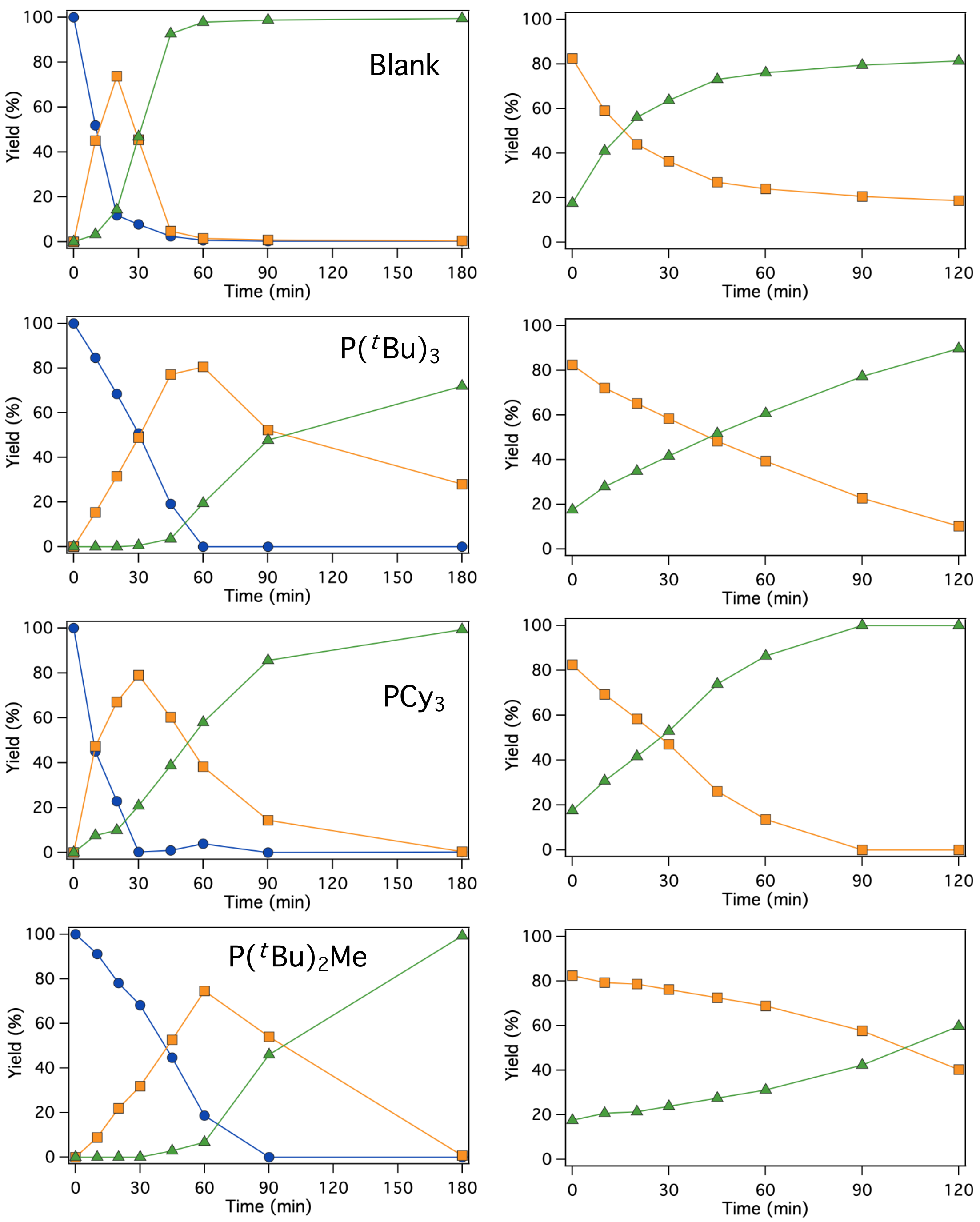
Blank
P(^{t}Bu)$_3$
PCy$_3$
P(^{t}Bu)$_2$Me
Yield (%)
Time (min)

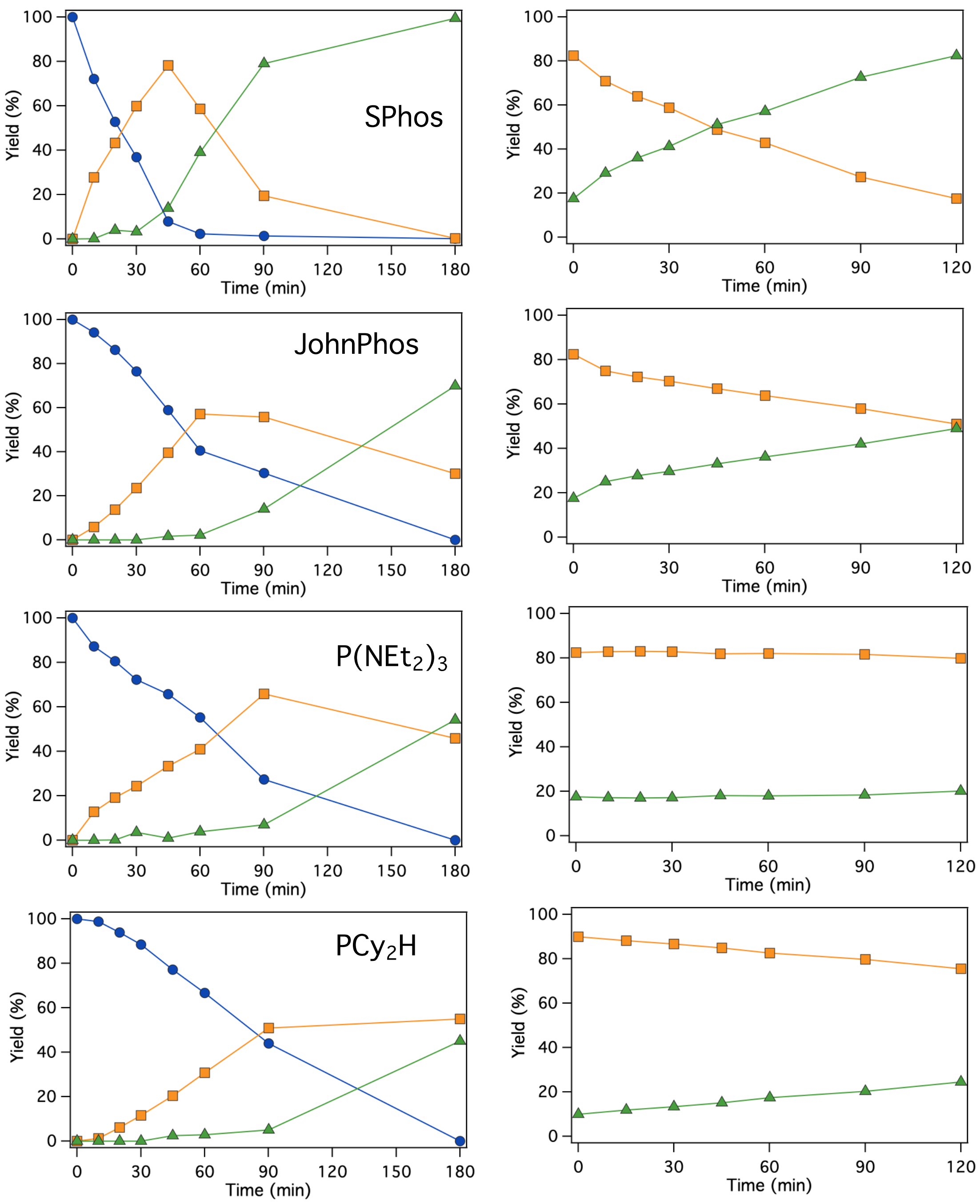
SPhos
JohnPhos
P(NEt2)3
PCy2H
Yield (%)
Time (min)

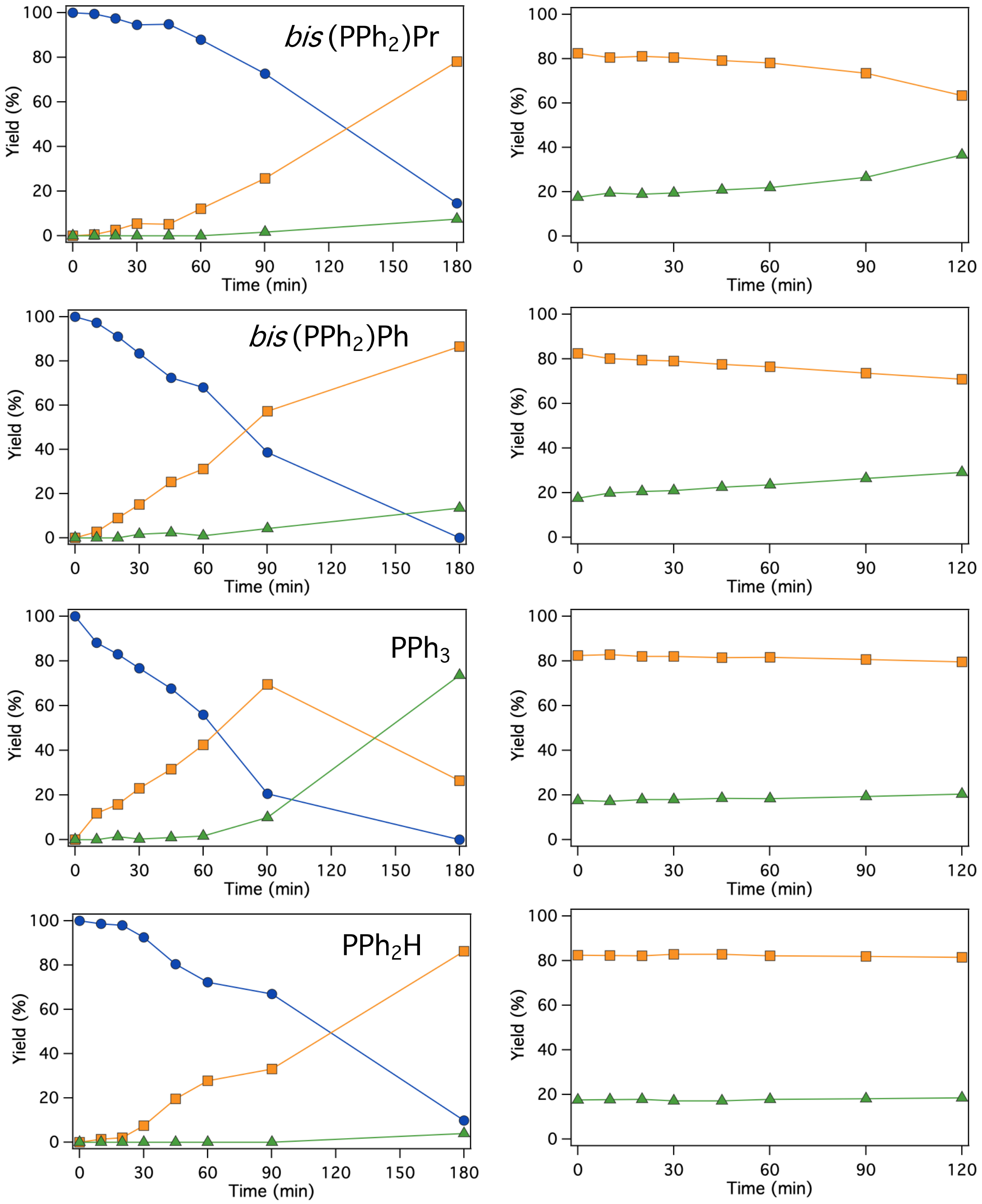

Figure S6. Kinetic profiles for the hydrogenation of **1** to **2** (left) and **2** to **3** (right) under 1 bar of H_2; blue = alkyne, orange = alkene, green = alkane.

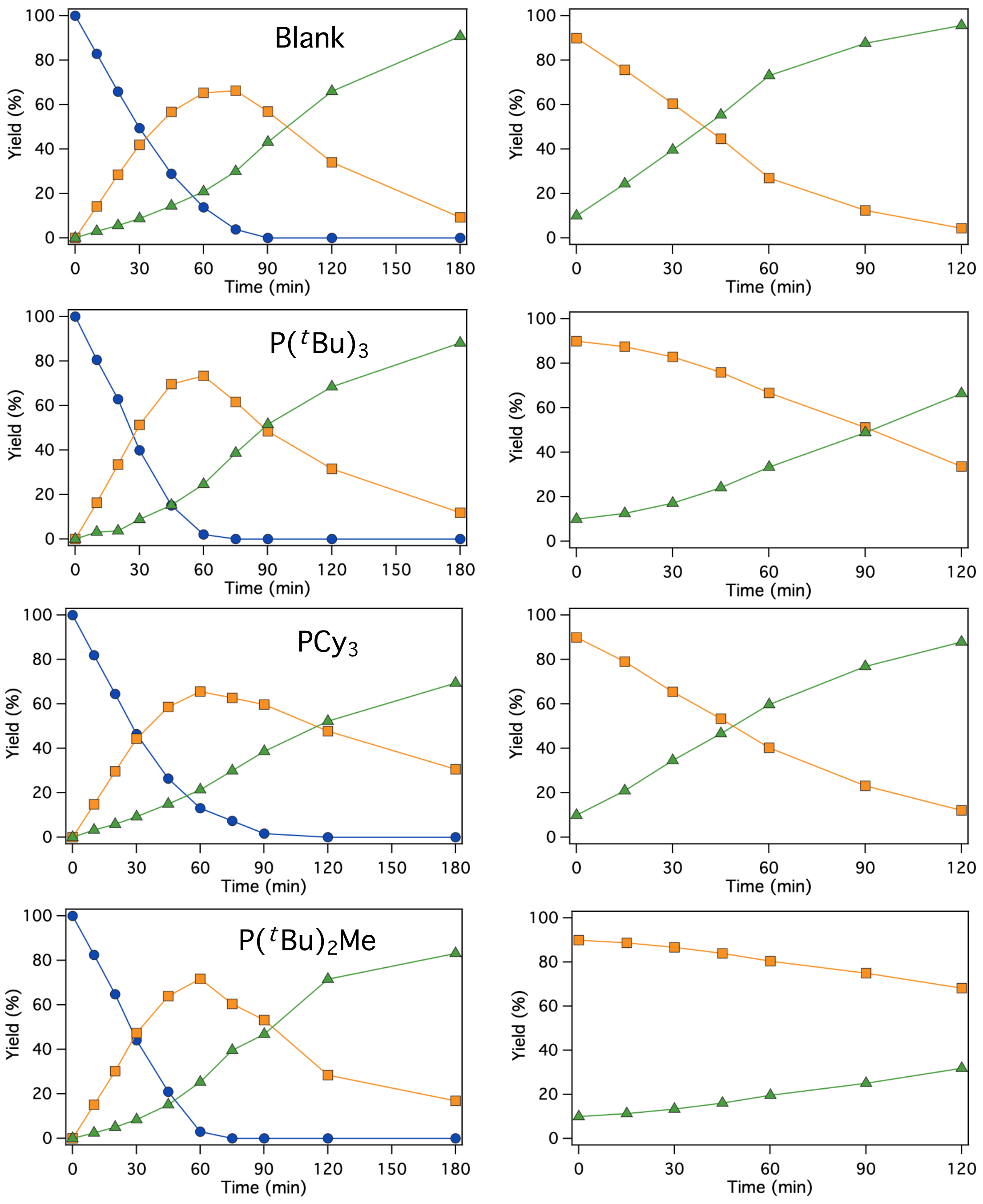
Blank
P(^{t}Bu)$_3$
PCy$_3$
P(^{t}Bu)$_2$Me
Yield (%)
Time (min)
0
20
40
60
80
100
0
30
60
90
120
150
180

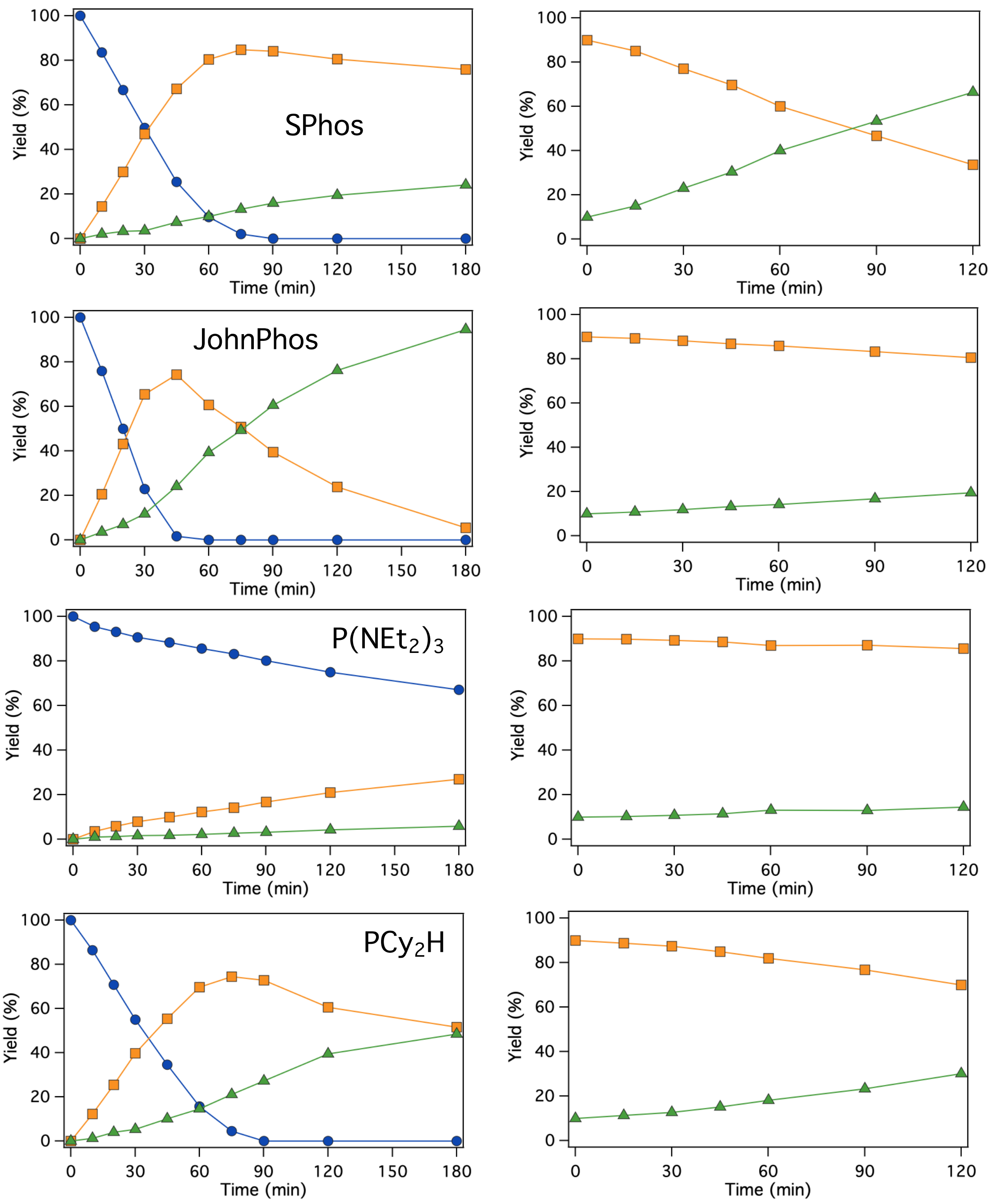
SPhos
JohnPhos
P(NEt2)3
PCy2H
Yield (%)
Time (min)

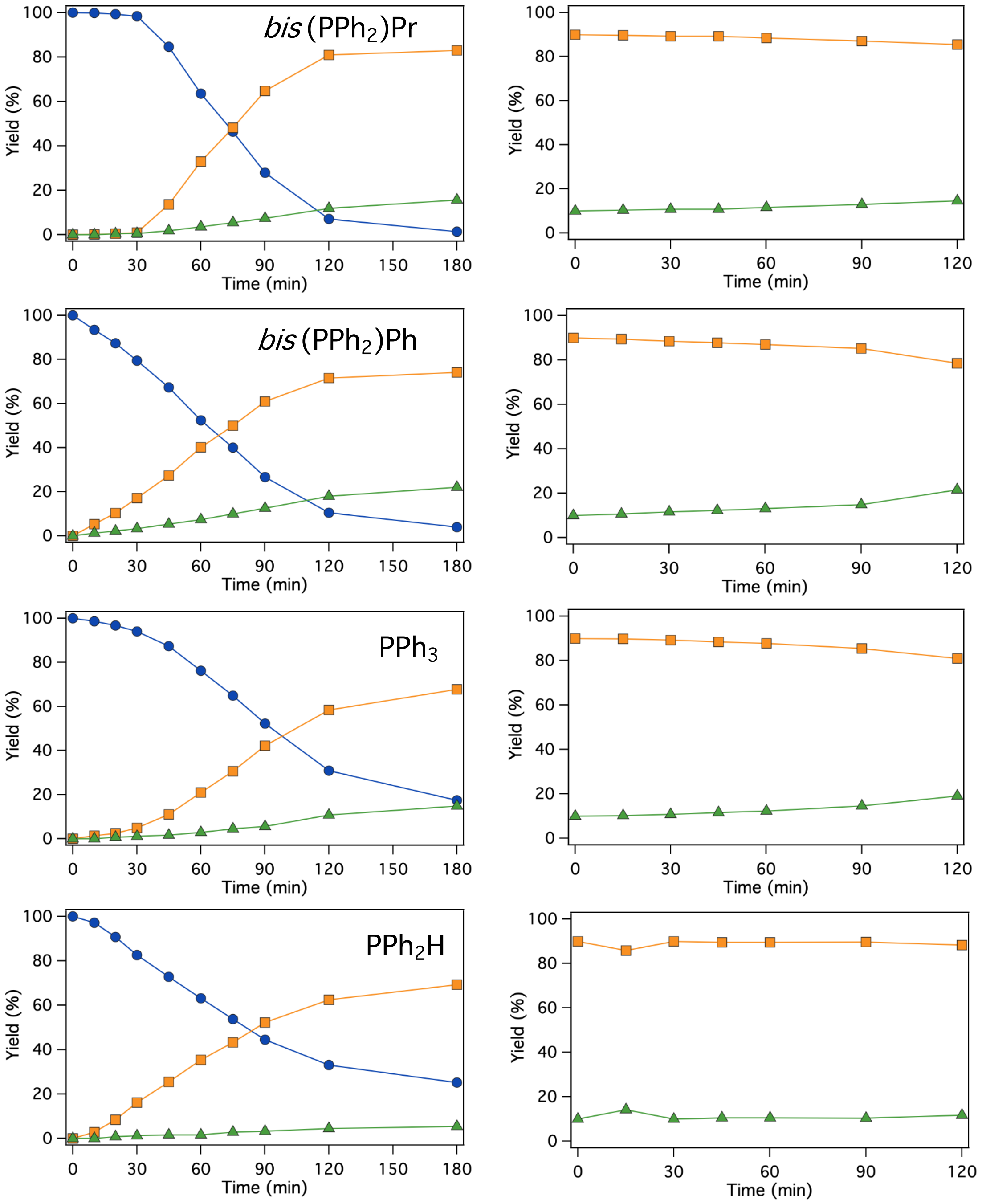

Figure S7. Kinetic profiles for the hydrogenation of **1** to **2** (left) and **2** to **3** (right) under 3 bar of H_2; blue = alkyne, orange = alkene, green = alkane.

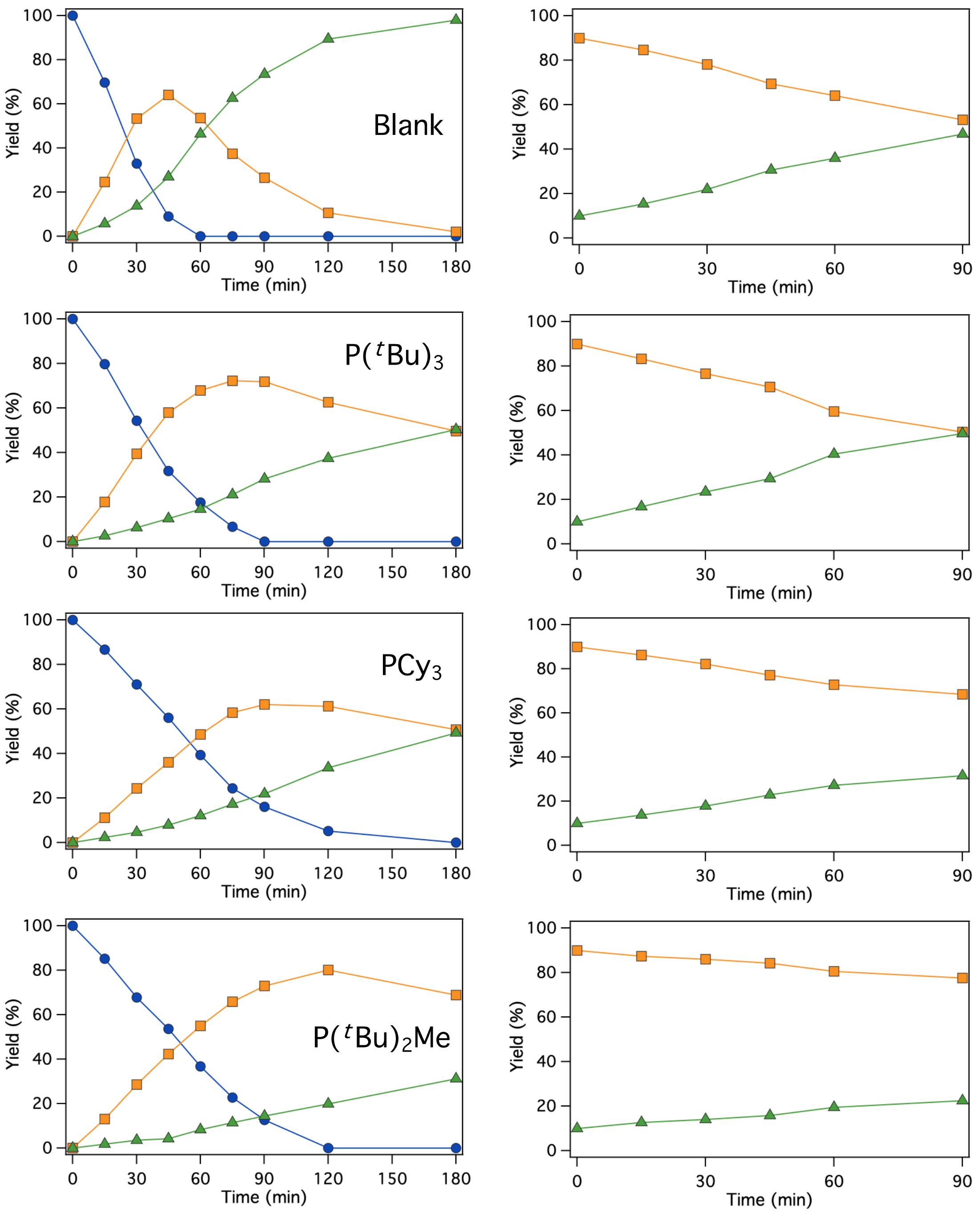
Blank
P(^{t}Bu)$_3$
PCy$_3$
P(^{t}Bu)$_2$Me
Yield (%)
Time (min)
0
20
40
60
80
100
0
30
60
90
120
150
180

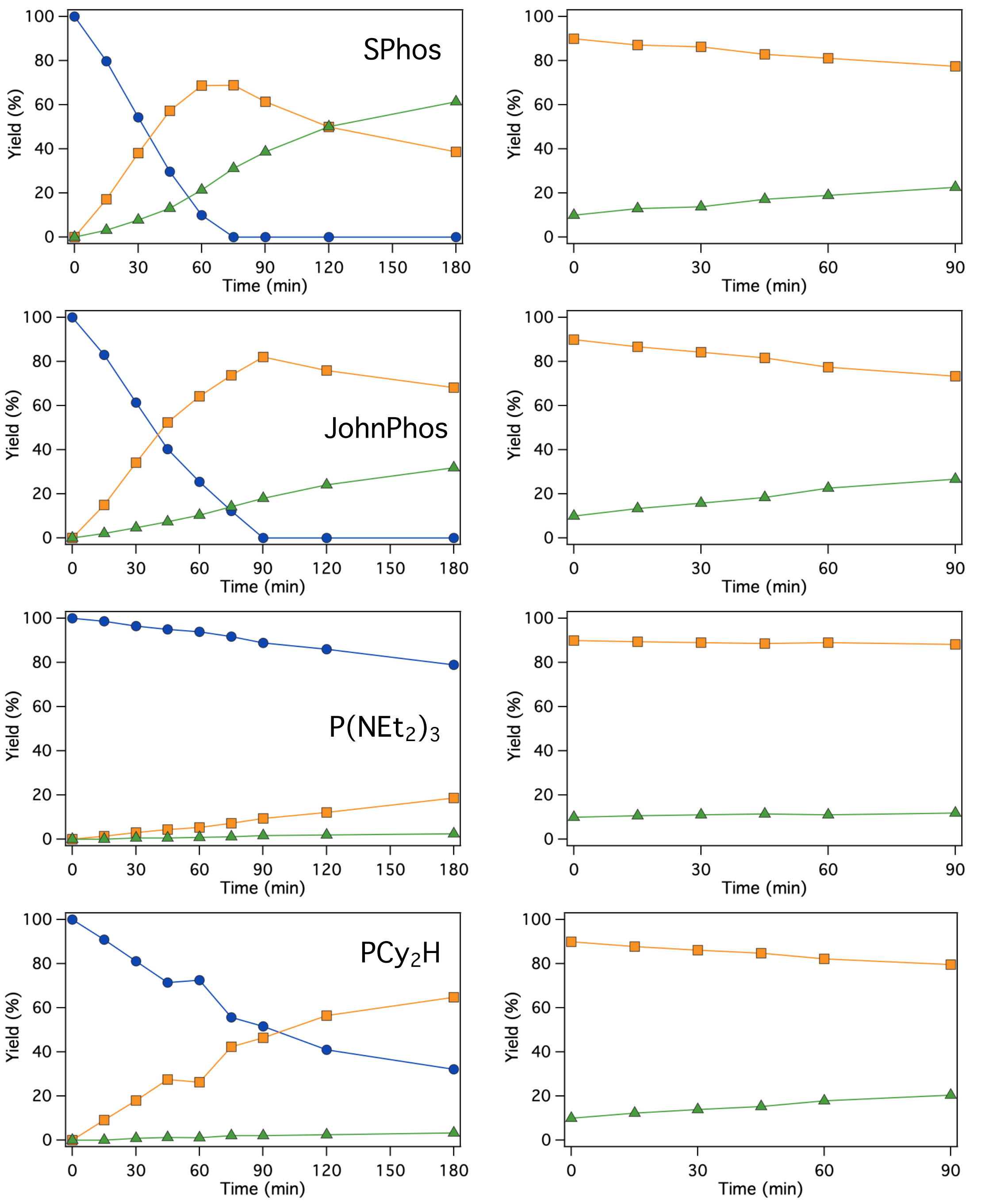
SPhos
JohnPhos
P(NEt2)3
PCy2H
Yield (%)
Time (min)
0
20
40
60
80
100
0
30
60
90
120
150
180

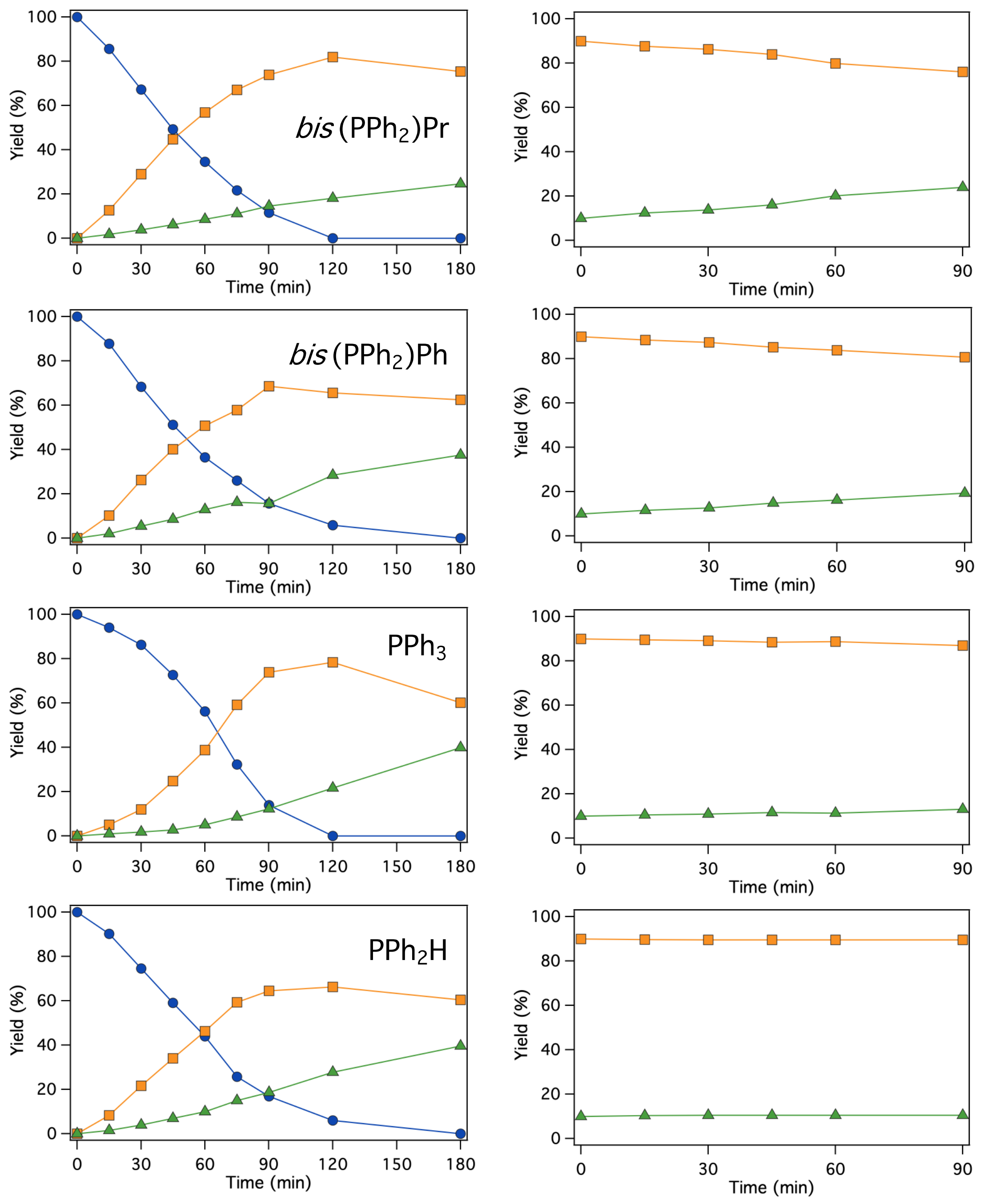

Figure S8. Kinetic profiles for the hydrogenation of **1** to **2** (left) and **2** to **3** (right) under 5 bar of H_2; blue = alkyne, orange = alkene, green = alkane.

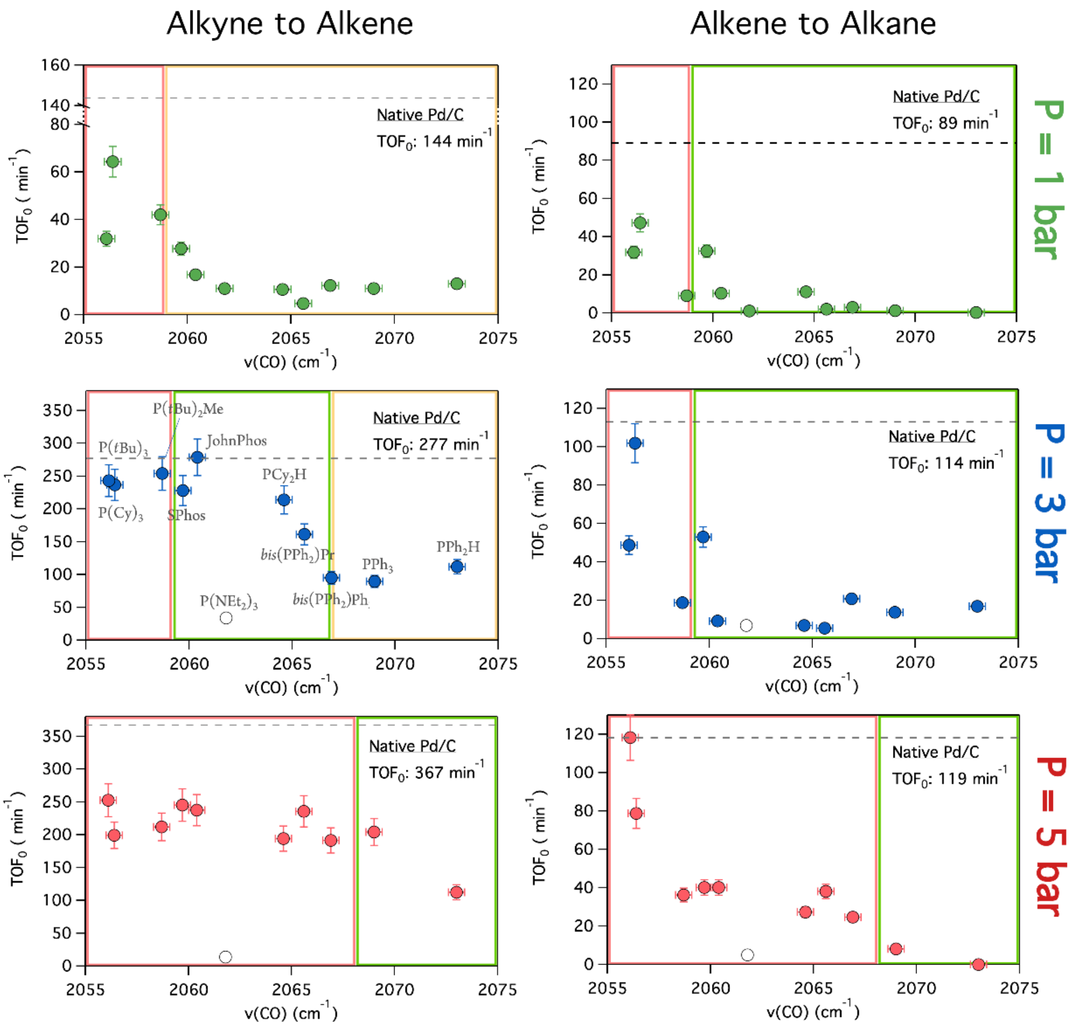

Figure S9. Correlations between initial turnover frequencies (TOF_0) in the alkyne to alkene (left) and alkene to alkane (right) hydrogenation reactions and the phosphine electronic properties, expressed in terms of ν(CO) (cm^{-1}) of the corresponding $Ni(CO)_3(PR_3)$ complex. The reactions were performed with Pd/C (0.03 mol%) at 1 bar of H_2 (top, green), 0.01 mol% Pd and 3 bar of H_2 (middle, blue), and 0.01 mol% Pd and 5 bar of H_2 (bottom, red); 1:20 ratio Pd to phosphine. Red Areas: alkene **2** can be hydrogenated under these conditions. Yellow areas: alkene **2** cannot be hydrogenated, low hydrogenation rates of alkyne **1**. Green Areas: alkene **2** cannot be hydrogenated, high hydrogenation rates of alkyne **1**. Vertical error bars represent ± 10% uncertainty, horizontal error bars represent the ± 0.3 cm^{-1} of uncertainty reported by Tolman et al.

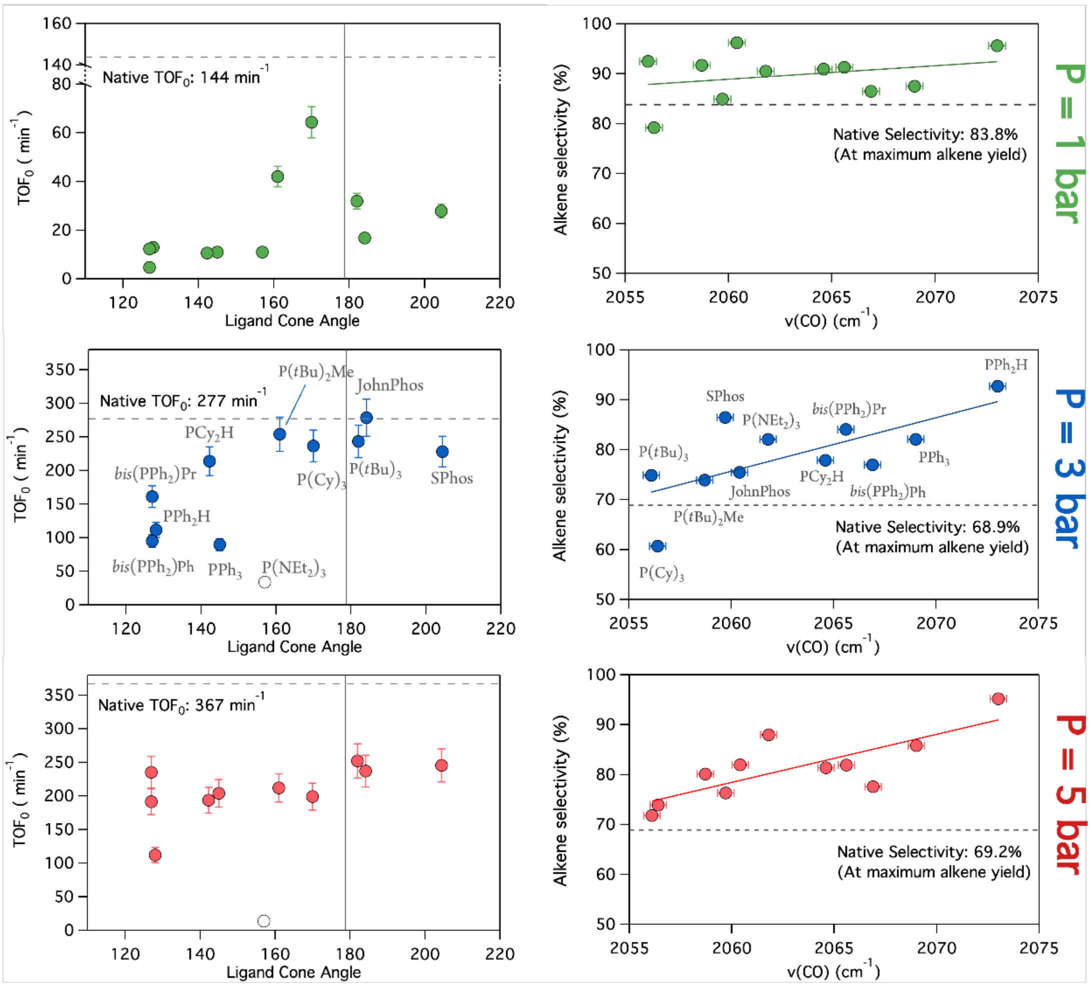

Figure S10. Left: correlations between initial turnover frequencies (TOF_0) in the **1** to **2** hydrogenation reactions and the Tolman cone angle of the phosphines. Right: correlations between the selectivity (at maximum alkene yield) towards the alkene and the electronic properties of the phosphines, expressed in terms of ν(CO) (cm^{-1}) of the corresponding $Ni(CO)_3(PR_3)$ complex. The reactions were performed with Pd/C (0.03 mol% Pd) at 1 bar of H_2 (top, green), 0.01 mol% Pd and 3 bar of H_2 (middle, blue), and 0.01 mol% Pd and 5 bar of H_2 (bottom, red); 1:20 molar ratio Pd : phosphine. Vertical error bars represent ± 10% uncertainty, horizontal error bars represent the ± 0.3 cm^{-1} of uncertainty reported by Tolman et al.

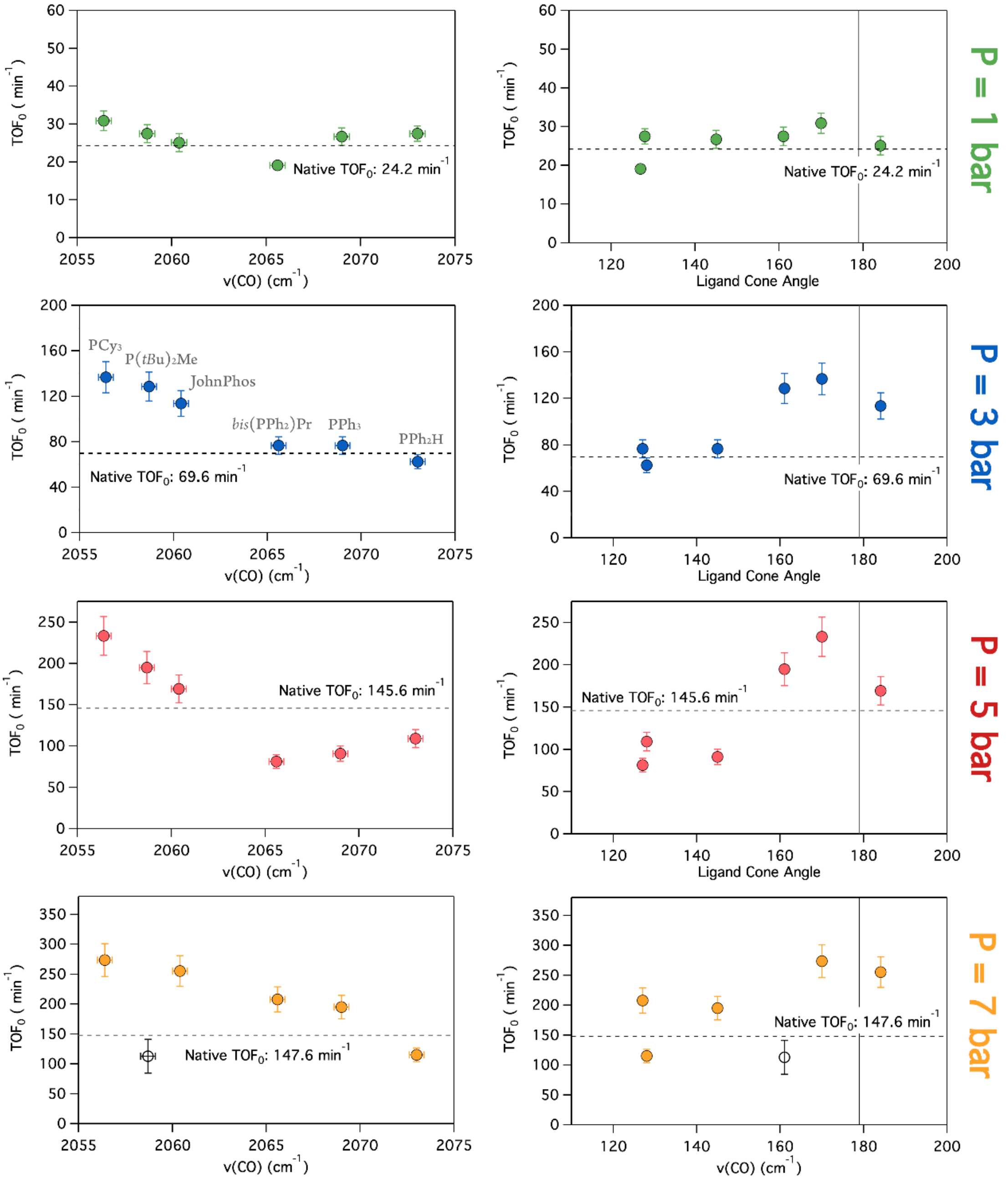

Figure S11. Left: correlations between turnover frequencies (TOF_0) in the hydrogenation reaction of 1-octyne (**4**) to the corresponding alkene and the phosphine electronic properties, expressed in terms of ν(CO) (cm^{-1}) of the corresponding $Ni(CO)_3(PR_3)$ complex. Right: correlations between TOF_0 of the hydrogenation reaction of alkyne **4** to the corresponding alkene and the phosphine steric properties, expressed in terms of the ligand cone angle (in deg.). The reactions were performed with Pd/C (0.03 mol% Pd) at 1 bar of H_2 (green), 0.01 mol% Pd and 3 bar of H_2 (blue), 0.01 mol% Pd and 5 bar of H_2 (red), and 0.01 mol% Pd and 7 bar of H_2 (orange); 1:20 ratio Pd to phosphine.

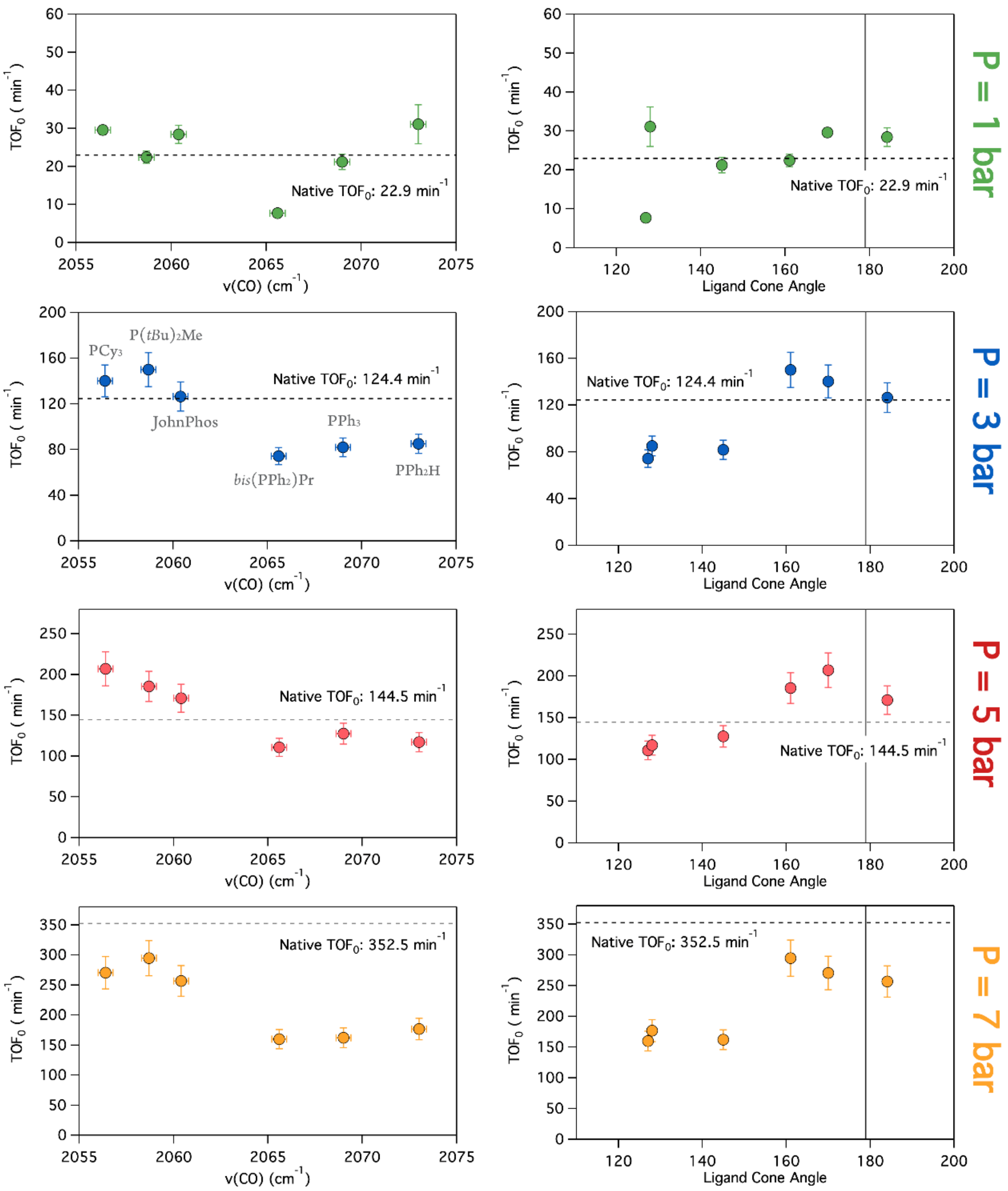

Figure S12. Left: correlations between turnover frequencies (TOF_0) in the hydrogenation reaction of phenylacetylene (**5**) to the corresponding alkene and the phosphine electronic properties, expressed in terms of ν(CO) (cm^{-1}) of the corresponding $Ni(CO)_3(PR_3)$ complex. Right: correlations between TOF_0 of the hydrogenation reaction of alkyne (**5**) to the corresponding alkene and the phosphine steric properties, expressed in terms of the ligand cone angle (in deg.). The reactions were performed with Pd/C (0.03 mol% Pd) at 1 bar of H_2 (green), 0.01 mol% Pd and 3 bar of H_2 (blue), 0.01 mol% Pd and 5 bar of H_2 (red), 0.01 mol% Pd and 7 bar of H_2 (orange); 1:20 ratio Pd to phosphine.

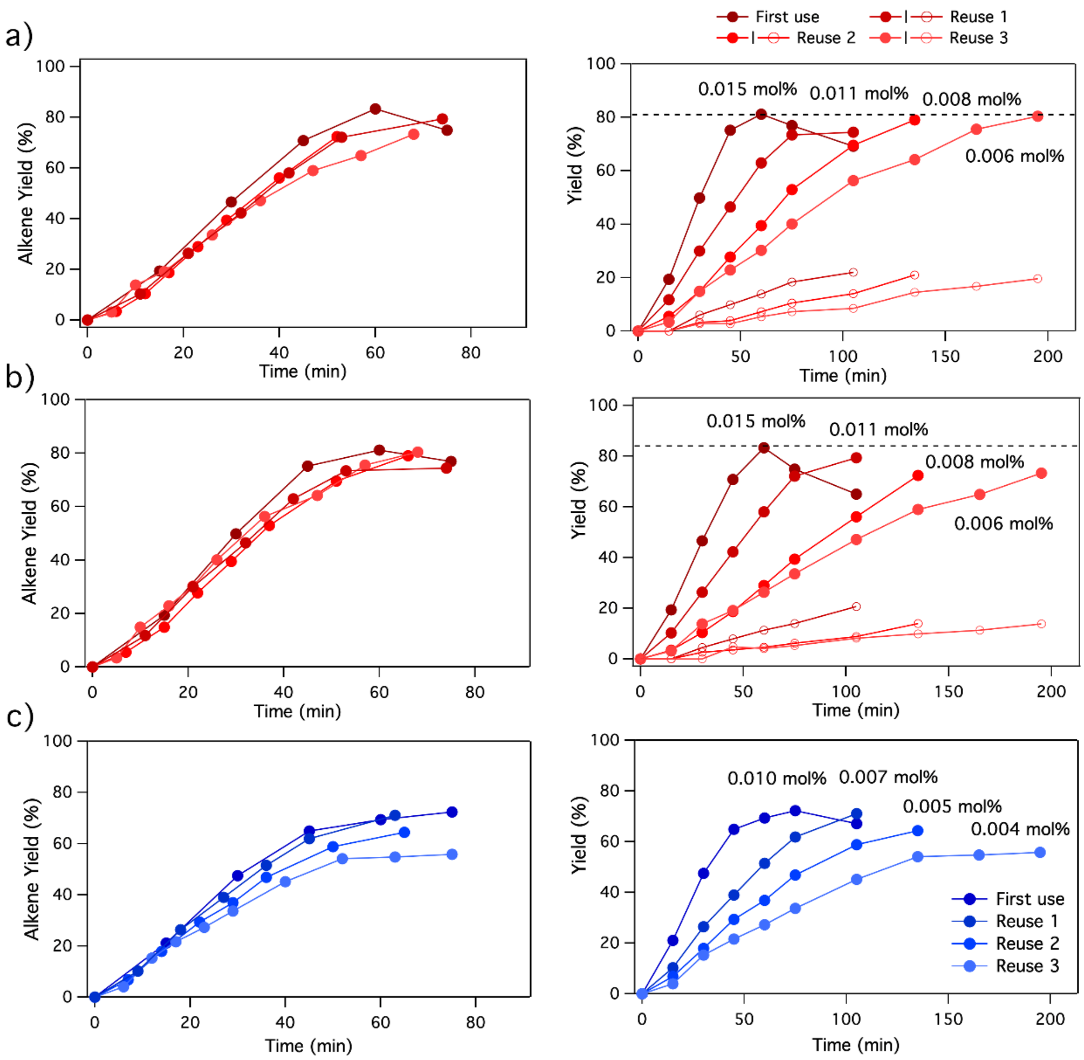

Figure S13. Reuse tests for the phosphine-modified Pd/C catalysts during the hydrogenation of alkyne **1**. Left: profiles normalized by catalyst mass; right: original kinetic profiles showing the mass (mol%) of Pd recovered from use to use. a) PPh_3 (20:1, P to Pd) added in the first use of the catalyst, 1 equivalent of phosphine was added to the catalyst in each further use. b) PPh_3 (20:1, P to Pd) added in the first use of the catalyst, without additional phosphine added in further uses. c) S-Phos (20:1, P to Pd) added in the first use of the catalyst, without additional phosphine added in further uses. All reactions were performed under 3 bar H_2, 0.6 M **1** and 30 ºC. Solid markers and hollow markers correspond to alkene and alkane yields, respectively.

Supporting Tables

Table S1. Phosphine ligand contributions to the ν(CO) vibrational frequency of the phosphines included in this work, as reported by Tolman et al.

Type	Number	Phosphine	R1	R2	R3	R1 x_i	R2 x_i	R3 x_i	$Ni(CO)_3L$ *ν*(CO), cm^{-1}	Tolman Cone Angle
PX_3	**P1**	$P(NEt_2)_3$	$N(CH_2\text{-}CH_3)_2$	$N(CH_2\text{-}CH_3)_2$	$N(CH_2\text{-}CH_3)_2$	1.9[a]	1.9[a]	1.9[a]	2061.8 ± 0.3	157.0
PR_3	**P2**	$P(^tBu)_3$	*tert*-Butyl	*tert*-Butyl	*tert*-Butyl	0	0	0	2056.1 ± 0.3	182.0
	**P3**	PCy_3	Cy	Cy	Cy	0.1	0.1	0.1	2056.4 ± 0.3	170.0
	**P4**	PPh_3	Ph	Ph	Ph	4.3	4.3	4.3	2069.0 ± 0.3	145.0
$P(R^1)_2R^2$	**P5**	$P(^tBu)_2Me$	*tert*-Butyl	*tert*-Butyl	Me	0	0	2.6	2058.7 ± 0.3	161.0
	**P6**	PCy_2H	Cy	Cy	H	0.1	0.1	8.3	2064.6 ± 0.3	142.3
	**P7**	PPh_2H	Ph	Ph	H	4.3	4.3	8.3	2073.0 ± 0.3	128.0
P_2R_4	**P8**	*bis*(PPh_2)Ph	Ph	Ph	Ph	4.3	4.3	2.15[b]	2066.9 ± 0.3	127.0[g]
	**P9**	*bis*(PPh_2)Pr	Ph	Ph	Propyl	4.3	4.3	0.9[c]	2065.6 ± 0.3	127.0
Buchwald's	**P10**	SPhos	Cy	Cy	2-MeO-1-1'-biPh	0.1	0.1	3.4[d]	2059.7 ± 0.3	204.4[h]
	**P11**	JohnPhos	*tert*-Butyl	*tert*-Butyl	1-1'-biPh	0	0	4.3[e]	2060.4 ± 0.3	184.1[i]

a) Estimated as NMe_2
b) Estimated as Ph/2
c) Estimated as Et/2
d) Estimated as (*o*-C_6H_4OMe)
e) Estimated as Ph
f) From Ref.[S5]
g) Assumed same angle as
9
h) From Ref.[S6]

$$v = 2056.1 + \sum_{i=1}^{3} x_i$$

Table S2. Numeric values of the TOF_0 extracted from the kinetic profiles and reported in Figures S6 to S8.

Type	Number	Phosphine	P(H_2) = 1 bar			P(H_2) = 3 bar			P(H_2)= 5 bar		
			TOF Alkene (min^{-1})	TOF Alkane (min^{-1})	Selectivity (%)	TOF Alkene (min^{-1})	TOF Alkane (min^{-1})	Selectivity (%)	TOF Alkene (min^{-1})	TOF Alkane (min^{-1})	Selectivity (%)
-	-	Blank	144.0	89.0	83.8	277.0	114.0	68.9	367.0	119.0	69.2
PX_3	**P1**	P(NEt_2)$_3$	11.0	1.1	90.5	34.0	6.9	82.1	13.7	5.0	88.0
PR_3	**P2**	P(^{t}Bu)$_3$	31.9	31.9	92.5	243.2	48.8	74.9	252.4	118.3	71.8
	**P3**	PCy_3	64.3	47.3	79.2	236.6	101.9	60.7	199.0	78.7	73.9
	**P4**	PPh_3	11.0	1.2	87.5	89.6	13.7	82.1	204.2	8.2	85.8
$P(R^1)_2R^2$	**P5**	P(^{t}Bu)$_2$Me	42.0	9.1	91.7	254.0	18.8	73.9	212.0	36.3	80.1
	**P6**	PCy_2H	10.6	11.2	90.9	213.9	6.8	77.9	194.0	27.4	81.4
	**P7**	PPh_2H	13.0	0.3	95.6	111.7	16.8	92.7	112.3	0.0	95.2
P_2R_4	**P8**	*bis*(PPh_2)Ph	12.3	3.0	86.5	95.2	20.8	77.0	191.4	24.7	77.6
	**P9**	*bis*(PPh_2)Pr	4.7	2.1	91.3	161.3	5.4	84.1	235.5	38.1	81.9
Buchwald's	**P10**	SPhos	27.8	32.5	84.9	228.1	52.9	86.5	245.4	40.1	76.3
	**P11**	JohnPhos	16.8	10.3	96.2	278.8	9.2	75.5	237.3	40.2	82.0

References

(S1) Maheut, G.; Hervieu, M.; Fernandez, C.; Montouillout, V.; Villemin, D.; Jaffrès, P. A. Palladium Complex Immobilised on Zirconium-Phosphite: Characterisation by 31P MAS NMR and TEM and Behaviour towards Reducing Agents. *J. Mol. Struct.* **2003**, *659* (1–3), 135–142. https://doi.org/10.1016/S0022-2860(03)00409-5.

(S2) Ye, E.; Tan, H.; Li, S.; Fan, W. Y. Self-Organization of Spherical, Core-Shell Palladium Aggregates by Laser-Induced and Thermal Decomposition of [Pd(PPh3)4]. *Angew. Chem. Int. Ed.* **2006**, *45* (7), 1120–1123. https://doi.org/10.1002/anie.200503408.

(S3) Moedritzer, K.; Maier, L.; Groenweghe, L. C. D. Phosphorus-31 Nuclear Magnetic Resonance Spectra of Phosphorus Compounds. *J. Chem. Eng. Data* **1962**, *7* (2), 307–310. https://doi.org/10.1021/je60013a043.

(S4) Sherman, R.; Birnbaum, H. K.; Holy, J. A.; Klein, M. V. Raman Studies of Hydrogen Vibrational Modes in Palladium. *Phys. Lett. A* **1977**, *62* (5), 353–355. https://doi.org/10.1016/0375-9601(77)90439-X.

(S5) Chadwick A., T. Steric Effects of Phosphorus Ligands in Organometallic Chemistry and Homogeneous Catalysis. *Chem. Rev.* **1977**, *77* (3), 314–348. https://doi.org/10.1021/cr60307a002

(S6) Jover, J.; Cirera, J. Computational Assessment on the Tolman Cone Angles for P-Ligands. *Dalt. Trans.* **2019**, *48* (40), 15036–15048. https://doi.org/10.1039/c9dt02876e.
